# The Atlantic salmon genome provides insights into rediploidization

**DOI:** 10.1038/nature17164

**Published:** 2016-04-18

**Authors:** Sigbjørn Lien, Ben F. Koop, Simen R. Sandve, Jason R. Miller, Matthew P. Kent, Torfinn Nome, Torgeir R. Hvidsten, Jong S. Leong, David R. Minkley, Aleksey Zimin, Fabian Grammes, Harald Grove, Arne Gjuvsland, Brian Walenz, Russell A. Hermansen, Kris von Schalburg, Eric B. Rondeau, Alex Di Genova, Jeevan K. A. Samy, Jon Olav Vik, Magnus D. Vigeland, Lis Caler, Unni Grimholt, Sissel Jentoft, Dag Inge Våge, Pieter de Jong, Thomas Moen, Matthew Baranski, Yniv Palti, Douglas R. Smith, James A. Yorke, Alexander J. Nederbragt, Ave Tooming-Klunderud, Kjetill S. Jakobsen, Xuanting Jiang, Dingding Fan, Yan Hu, David A. Liberles, Rodrigo Vidal, Patricia Iturra, Steven J. M. Jones, Inge Jonassen, Alejandro Maass, Stig W. Omholt, William S. Davidson

**Affiliations:** 1 Department of Animal and Aquacultural Sciences, Centre for Integrative Genetics (CIGENE), Norwegian University of Life Sciences, Ås NO-1432, Norway; 2 Department of Biology, University of Victoria, Victoria, British Columbia, V8W 3N5 Canada; 3 J. Craig Venter Institute, 9704 Medical Center Drive, Rockville, 20850 Maryland USA; 4 Department of Chemistry, Biotechnology and Food Science, Norwegian University of Life Sciences, Ås NO-1432, Norway; 5 Department of Plant Physiology, Umeå Plant Science Centre, Umeå University, Umeå, 90187 Sweden; 6 Institute for Physical Sciences and Technology, University of Maryland, College Park, Maryland, 20742-2431 USA; 7 Department of Molecular Biology, University of Wyoming, Laramie, Wyoming, 82071 USA; 8 Center for Computational Genetics and Genomics, Temple University, Philadelphia, 19122-6078 Pennsylvania USA; 9 Department of Biology, Temple University, Philadelphia, 19122-6078 Pennsylvania USA; 10 Center for Mathematical Modeling, University of Chile, Santiago 8370456, Chile; 11 Center for Genome Regulation, University of Chile, Santiago 8370415, Chile; 12 Medical Genetics, Oslo University Hospital and University of Oslo, Oslo, NO-0424 Norway; 13 Department of Virology, Norwegian Veterinary Institute, Oslo, NO-0454 Norway; 14 Department of Biosciences, Centre for Ecological and Evolutionary Synthesis (CEES), University of Oslo, Oslo, NO-0316 Norway; 15 CHORI, Oakland, 94609 California USA; 16 AquaGen, Trondheim, NO-7462 Norway; 17 Nofima, Tromsø, NO-9291 Norway; 18 National Center for Cool and Cold Water Aquaculture, ARS-USDA, Kearneysville, 25430 West Virginia USA; 19 Beckman Genomics, Danvers, Massachusetts, 01923 USA; 20 Courtagen Life Sciences, Woburn, 01801 Massachusetts USA; 21 BGI-Shenzhen, Shenzhen, 518083 China; 22 Department of Biology, Laboratory of Molecular Ecology, Genomics, and Evolutionary Studies, University of Santiago, Santiago 9170022, Chile; 23 Faculty of Medicine, University of Chile, Santiago 8380453, Chile; 24 Genome Sciences Centre, BC Cancer Agency, Vancouver, British Columbia, V5Z 4S6 Canada; 25 Department of Molecular Biology and Biochemistry, Simon Fraser University, Burnaby, V5A 1S6 British Columbia Canada; 26 Department of Informatics, University of Bergen, Bergen, NO-6020 Norway; 27 Department of Biology, Centre for Biodiversity Dynamics, NTNU - Norwegian University of Science and Technology, Trondheim, NO-7491 Norway

**Keywords:** Genome evolution, Genome

## Abstract

The whole-genome duplication 80 million years ago of the common ancestor of salmonids (salmonid-specific fourth vertebrate whole-genome duplication, Ss4R) provides unique opportunities to learn about the evolutionary fate of a duplicated vertebrate genome in 70 extant lineages. Here we present a high-quality genome assembly for Atlantic salmon (*Salmo salar*), and show that large genomic reorganizations, coinciding with bursts of transposon-mediated repeat expansions, were crucial for the post-Ss4R rediploidization process. Comparisons of duplicate gene expression patterns across a wide range of tissues with orthologous genes from a pre-Ss4R outgroup unexpectedly demonstrate far more instances of neofunctionalization than subfunctionalization. Surprisingly, we find that genes that were retained as duplicates after the teleost-specific whole-genome duplication 320 million years ago were not more likely to be retained after the Ss4R, and that the duplicate retention was not influenced to a great extent by the nature of the predicted protein interactions of the gene products. Finally, we demonstrate that the Atlantic salmon assembly can serve as a reference sequence for the study of other salmonids for a range of purposes.

**Supplementary information:**

The online version of this article (doi:10.1038/nature17164) contains supplementary material, which is available to authorized users.

## Abstract (web summary)

The genome sequence is presented for the Atlantic salmon (Salmo salar), providing information about a rediploidization following a salmonid-specific whole-genome duplication event that resulted in an autotetraploidization.

**Supplementary information:**

The online version of this article (doi:10.1038/nature17164) contains supplementary material, which is available to authorized users.

## A window on salmonid genome evolution

William Davidson and colleagues report sequencing and assembly of the Atlantic salmon genome, which they demonstrate as a useful reference to also improve the genome assembly of other salmanoids. Their analyses provide insights into duplicate retention patterns across two rounds of whole-genome duplication that have occurred in this lineage.

**Supplementary information:**

The online version of this article (doi:10.1038/nature17164) contains supplementary material, which is available to authorized users.

## Main

The 22,000-year-old cave painting of an Atlantic salmon (*Salmo salar*) near the Vézère River in France is a reminder of our fascination with, and dependence on, Atlantic salmon throughout human history. Atlantic salmon belongs to the salmonid lineage which comprises 11 genera, with at least 70 species that exhibit a wide range of ecological adaptations and use a variety of marine and freshwater life history strategies^1^. Salmonids hold important positions as socially iconic species and economic resources within aquaculture, wild fisheries and recreational sport fisheries. Moreover, they serve as key indicator species of the health of North Atlantic and Pacific coastal and river ecosystems.

**Figure 1:**
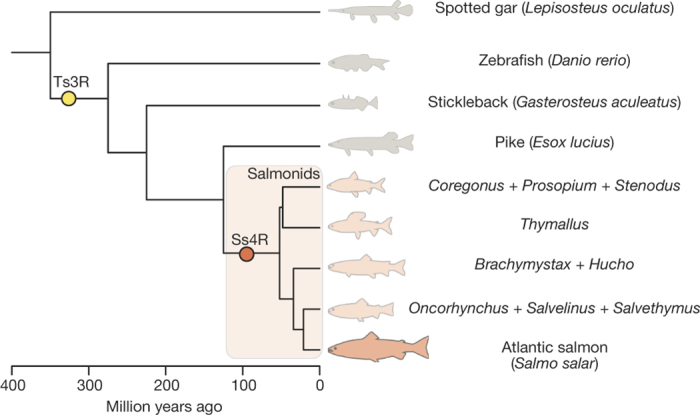
Phylogenetic relationship of salmonids and relevant teleost lineages. Divergence ages for salmonids are taken from ref. 8 and older divergences from ref. 7. *Parahucho* is not included in the figure due to uncertainty of its phylogenetic position. Ages do not represent the exact point estimates from the respective studies. Yellow and red circles represent the teleost specific whole genome duplication (Ts3R) and salmonid-specific whole genome duplication (Ss4R), respectively. PowerPoint slide

All teleosts share at least three rounds of whole-genome duplication (WGD), 1R and 2R before the divergence of lamprey from the jawed vertebrates^2^, and a third teleost-specific WGD (Ts3R) at the base of the teleosts ~320 million years ago (Mya)^3,4,5^. Very little is known about the mechanisms of genomic and chromosomal reorganization after WGD in vertebrates because the 1R, 2R and Ts3R occurred so long ago that few clear signatures of post-WGD reorganization events remain. In contrast, a fourth WGD (the Ss4R salmonid-specific autotetraploidization event) occurred in the common ancestor of salmonids ~80 Mya after their divergence from Esociformes ~125 Mya^6,7,8^ (Fig. 1), and the continued presence of multivalent pairing at meiosis and evidence of tetrasomic inheritance in salmonid species suggests that diploidy is not yet fully re-established^6,9,10^. Salmonids thus appear to provide an unprecedented opportunity for studying vertebrate genome evolution after an autotetraploid WGD^11,12^ over a time period that is long enough to reveal long-term evolutionary patterns, but short enough to give a high-resolution picture of the process. In addition, they provide an excellent setting for contextualizing genome evolution with a dramatic post-WGD species radiation and intricate adaptations to a whole range of life history regimes.

Here we present a high-quality reference genome assembly of the Atlantic salmon, and use it to describe major patterns characterizing the post-Ss4R salmonid genome evolution over the past 80 million years (Myr). Our results challenge the recent claim that rediploidization in salmonids has been a gradual process unlinked to significant genome rearrangements^13^. They also challenge current views about the relative importance of sub- and neofunctionalization in vertebrate genomes (reviewed in ref. 14), and the importance of dosage balance as a gene duplicate retention mechanism^15^.

## Genome characterization

**Figure 2:**
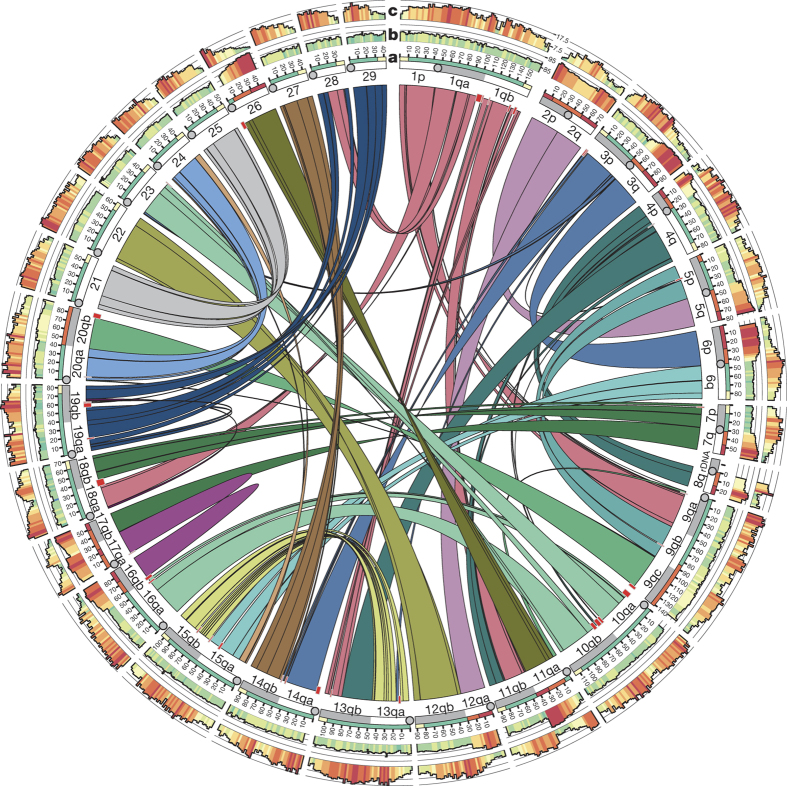
The duplicated Atlantic salmon genome. Homeologous regions in the Atlantic salmon genome subdivided into 98 collinear blocks along the 29 European Atlantic salmon chromosomes. Red rectangles represent blocks of sequence without identifiable duplicated regions elsewhere in the genome. **a**, This track shows grouping of salmon sequence into regions; red = high (>95% sequence similarity), orange = elevated (90–95% sequence similarity), green = low (~87% sequence similarity), yellow = telomeric regions (10 Mb) characterized by highly elevated male recombination (see ref. 10). **b**, This track shows genomic similarity (in 1 Mb intervals) between duplicated regions (red = high, yellow = medium, green = low sequence similarity). **c**, Ths track shows frequency of Tc1-*mariner* transposon elements in the Atlantic salmon genome. PowerPoint slide

The Atlantic salmon reference genome assembly (GenBank: GCA_000233375.4) adds up to 2.97 gigabases (Gb) with a ctgN50 = 57.6 kb, which is consistent with genome size estimates^16^. Linkage mapping was used to position and orient 9,447 scaffolds (scfN50 = 2.97 megabases (Mb)), representing 2.24 Gb, into 29 single chromosome sequences (Supplementary Table 4). Most scaffolds not anchored to chromosomes consist of repetitive sequences. The 58–60% repeat content of Atlantic salmon is among the highest found in any vertebrate^17^. The single largest class of transposable elements is the Tc1-*mariner* family, representing 12.89% of the genome (Supplementary Information section 3). Tc1-*mariner* transposons tend to occur in centromeric regions (Fig. 2, track c), as reported in other species^18^.

Annotation of gene structures using RNA sequencing (RNA-seq) and expressed sequence tags (ESTs) identified 46,598 genes classified as non-repeat associated loci with sequence similarity support from the PFAM database, and/or zebrafish and stickleback annotations (Supplementary Table 11). Functional annotation identified a final set of 37,206 high-confidence protein-coding gene loci that have been assigned a putative functional annotation based on homology within the SwissProt database. Ninety-five per cent of the 498,245 public ESTs, and 98.3% of the identified loci were mapped to the 29 chromosome sequences, indicating a nearly complete representation of the protein-coding genome (Supplementary Information section 1.5).

## Post-Ss4R rediploidization characteristics

The return of a duplicated genome from tetrasomic to disomic inheritance relies on the obstruction of quadrivalent pairing during meiotic cell division. Large chromosome rearrangements through chromosome fusions, fissions, deletions or inversions strongly disrupt the possibility for homeologous pairing (the pairing of homeologue duplicates arising from a WGD)^19,20^. As extensive collinear blocks that include the telomere for at least one of the chromosome pairs is a diagnostic for current or recent multivalent pairing due to sequence homogenization (reviewed in ref. 21), we predicted that there would be an inverse relationship between homeologous sequence similarity and chromosome rearrangements in the duplicated blocks.

To test this prediction, we identified and analysed 98 homeologous (duplicated) blocks with high collinearity by aligning Atlantic salmon chromosome sequences against each other (Supplementary Information section 2). The 98 blocks (196 regions) account for 2.11 Gb (94.4%) of chromosome-anchored sequence (Fig. 2, Supplementary Table 6). A large proportion of homeologous blocks, representing roughly 573 Mb (25.6% of the chromosome-positioned sequence), had a sequence similarity >90%. These regions were clustered within seven pairs of chromosome arms (2p–5q, 2q–12qa, 3q–6p, 4p–8q, 7q–17qb, 11qa–26, 16qb–17qa, and to some extent 9qc–20qb and 5p–9qb (Fig. 2)), and are all characterized by large collinear blocks including the telomere within at least one of the chromosome pairs. Previous studies in salmonids have claimed that at least one metacentric chromosome must be involved to provide the stability required for the formation of multivalents and homeologous pairing^22^. Our findings for regions 11qa–26 and 16qb–17qa indicate that this is not a strict necessity. Notably, increased read alignment depth and shorter scaffolds were characteristic of regions exceeding 95% similarity, representing 210 Mb (9.4% of the chromosome-positioned sequence), suggesting assembly collapse (Fig. 2, Supplementary Information section 1.5).

**Extended Data Figure 1:**
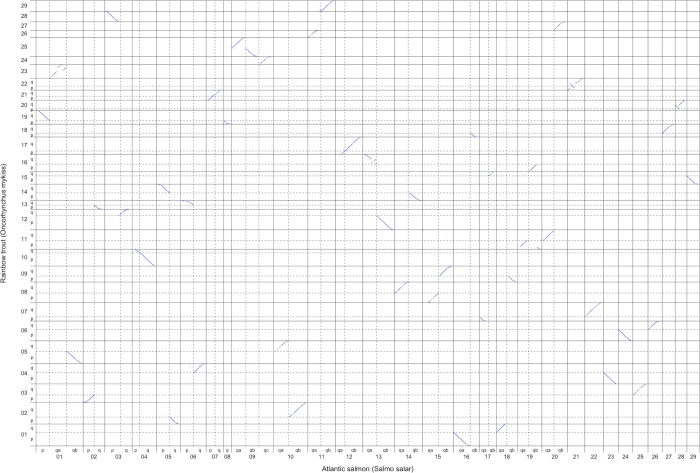
Atlantic salmon and rainbow trout comparative map. Alignment of Atlantic salmon (*Salmo salar*) and rainbow trout (*Oncorhynchus mykiss*) chromosome sequences using LASTZ demonstrates conservation of large collinear syntenic blocks between the two species.

Without exception, duplicated regions exhibiting rearrangements at telomeres in the form of inversions, translocations or larger deletions all displayed a sequence similarity of ~87%. This clear correspondence between the degree of intra-block sequence similarity and blocks predicted to still participate in tetrasomic inheritance (or recently have done so) suggests that up to 25% of the salmon genome experienced delayed rediploidization after the initial large chromosome rearrangements, and that as much as 10% of the genome may still retain residual tetrasomy (Supplementary Table 7). The large and highly collinear blocks of shared synteny between Atlantic salmon and rainbow trout (Extended Data Fig. 1) imply that these rearrangements must have taken place before the split of the two lineages. This is also supported by combined genome mapping and karyotyping studies in other members of the Salmoninae subfamily, documenting conservation of large blocks embracing whole chromosome arms^22^.

**Figure 3:**
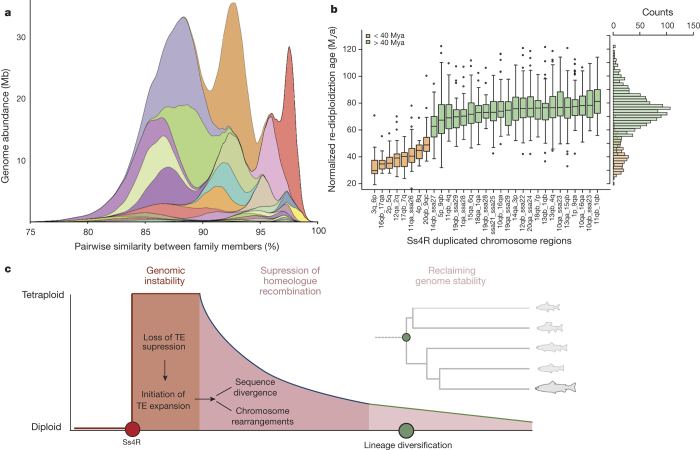
Post-Ss4R rediploidization. **a**, Fig. 3a shows a significant and ongoing expansion of transposable elements from the Tc1-*mariner* superfamily with major peaks at an average of 87%, 93% and 98% similarity between family members. The colours correspond to the same colours as in the box plot in Extended Data Fig. 5. **b**, Age estimates of the time from homeologue divergence to *Salmo–Oncorhynchus* divergence for each individual homeologous region. Only chromosome regions with >10 gene trees were included. **c**, A three-step hypothetical model of post-Ss4R rediploidization (widths of model compartments do not reflect actual time scales). The green circle indicates the beginning of the salmonid radiation. PowerPoint slide

**Extended Data Figure 2:**
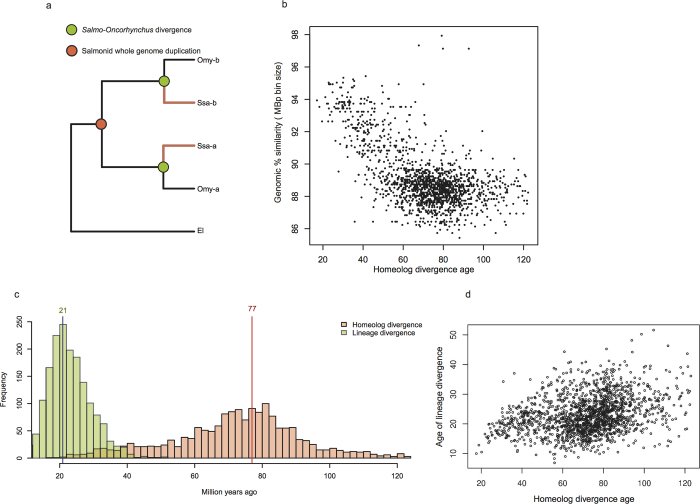
Dating or Ss4R rediploidization. **a**, Schematic representation of a gene tree topology reflecting rediploidization of Ss4R homeologues before *Salmo–Oncorhynchus* divergence. **b**, Correlation between genomic similarity in 1 Mb windows and Ss4R rediploidization (that is, divergence) age. **c**, Distribution of *Salmo–Oncorhynchus* divergence age and Ss4R divergence age from time calibrated gene trees estimated with BEAST. Modes of each distribution are indicated with a vertical line. **d**, Correlation between estimated age of *Salmo–Oncorhynchus* divergence and Ss4R divergence age.

To scrutinize this further, we analysed a set of 2,487 gene trees from orthologous gene sets containing putative homeologous pairs for both Atlantic salmon and rainbow trout (*Oncorhynchus mykiss*) (Supplementary Information section 5). As this analysis required calibration against an outgroup, we included only homeologous pairs having an orthologue in the Northern pike (*Esox lucius*), a member of the closest related diploid sister-group to salmonids^23^. Our results suggest ~100–80 Mya as a lower boundary for the Ss4R and that the *Salmo–Oncorhynchus* divergence occurred ~21 Mya (Fig. 3b; Extended Data Fig. 2c and Supplementary Information section 6), in agreement with recent age estimates^8,13^. Interestingly, analysis of asymmetry in coding sequence evolution between homeologues showed that a major part of the sequence divergence happened since the *Salmo–Oncorhynchus* split, suggesting a considerable temporal decoupling between the Ss4R event and sequence divergence of the Ss4R duplicates (Supplementary Information section 6). Moreover, our molecular dating results suggest that the majority of the Ss4R duplicates returned to disomic inheritance in a common ancestor of all salmonids before ~60 Mya (Fig. 3c). The results from the gene tree analysis are thus consistent with the data on homeologous sequence similarity (Extended Data Fig. 2b), strongly suggesting that large genomic reorganizations have been instrumental for the rediploidization process following the Ss4R. Our findings thus challenge one of the main conclusions from the recent sequencing of the rainbow trout genome, which suggested that rediploidization in salmonids has been a gradual process unlinked to significant genome rearrangements^13^.

Considering possible mechanisms underlying these large genomic reorganizations, the distribution of major transposon families in the Atlantic salmon genome suggests transposable element expansion in an ancestral salmonid before the chromosome fusions occurring in the Atlantic salmon lineage (Fig. 2, track c). The 85% sequence divergence among a large number of transposon family members is comparable to the lower boundary of homeologue block similarity (~87%). Assuming comparable neutral clock-like sequence divergence, this correspondence is consistent with a burst of repeat expansions coinciding with the initiation of rediploidization post-Ss4R (Fig. 3a and Extended Data Fig. 2b and Supplementary Information section 6.2). As large-scale expansion and movement of transposable elements are known to increase under genomic stress^24^, this may suggest that Ss4R caused transposable element expansion by compromising regulatory processes responsible for transposon policing. This expansion might in turn have been a major determinant for driving the genome towards a diploid state through enhanced homeologue sequence divergence and large chromosome rearrangements due to ectopic transposable element recombination and chromosomal breakage causing non-homologous end-joining^25^ (Fig. 3c).

## Duplicate retention—patterns and mechanisms

To assess the evolutionary fates of duplicated genes in the salmon genome, we analysed patterns of Ss4R duplicate retention and functional divergence of protein-coding genes within the 98 homeologous blocks. Considering that we find very little evidence for gene loss through fractionation^26^, and that in 56% of the 9,162 singletons we were able to identify a pseudogenized homeologue gene fragment in an expected position (Supplementary Information section 4 and Supplementary Table 11), pseudogenization appears to be the predominant mechanism underlying Ss4R duplicate loss.

To contrast the Ss4R with the 240 Myr older Ts3R duplicate retention patterns, we analysed duplicate retention patterns in teleost gene family trees (ref. 27; Supplementary Information section 8). This revealed that 20% of the Ts3R and 55% of the Ss4R duplicates are retained as two functional copies in Atlantic salmon. In comparison, 12–24% of duplicated genes derived from the Ts3R event have been retained in other extant teleost fish lineages (reviewed in ref. 28), and the retention 75 Myr post-Ts3R has been estimated to have been about 40%^3,29^. Considering the uncertainty attached to such estimates, the post-Ss4R temporal retention profile of Atlantic salmon is arguably quite similar to that of other teleosts post-Ts3R, indicating that mechanisms responsible for duplicate retention in Atlantic salmon may be generic.

**Extended Data Figure 3:**
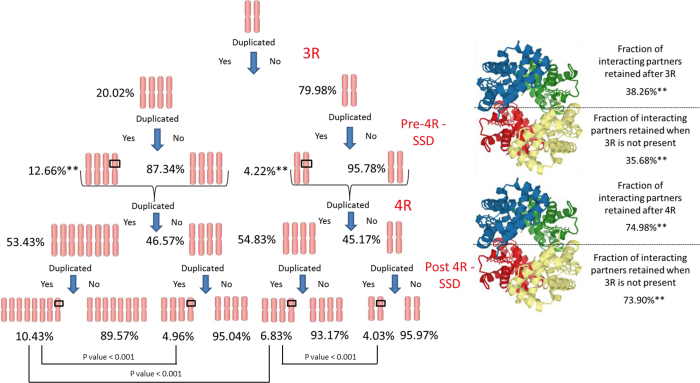
Duplication count analysis and interacting partner co-retention. The duplication process is depicted with the associated conditional probabilities for each type of duplication based upon a sampling of gene families that includes *Lepisosteus oculatus*. WGD events occur at both the Ts3R and Ss4R levels with individual gene duplications occurring at Pre-Ss4R–SSD and Post-Ss4R–SSD. Pre-Ss4R conditional probabilities are only dependent on Ts3R WGD being present and Ss4R WGD are only conditional on a Ts3R WGD being present. Retained interacting partners were determined from the STRING database^48^ as partners with (binding) physical interaction. Interacting partners were determined based on being retained after the same Ts3R WGD or a Ss4R WGD as the query sequence and having a homologue in *Danio rerio*. Two asterisks indicate significance at α < 0.001 (Bonferroni corrected) based on a two-proportion pooled *z*-test from a binomial distribution.

Surprisingly, Atlantic salmon genes that were retained as duplicates after the Ts3R event were not more likely to be retained after the Ss4R (Extended Data Fig. 3; Supplementary Information section 8). The predominantly independent probabilities of retention suggest a complex interplay of processes, different evolutionary drivers of duplicate retention, or a largely neutral and stochastic nonfunctionalization process following the Ts3R and Ss4R events. Interestingly, we observed enhanced retention of non-WGD gene duplicates (older or younger than the Ss4R event) when the WGD (both Ts3R and Ss4R) duplicates also had been retained (*P* < 0.001; Supplementary Information section 8).

**Figure 4:**
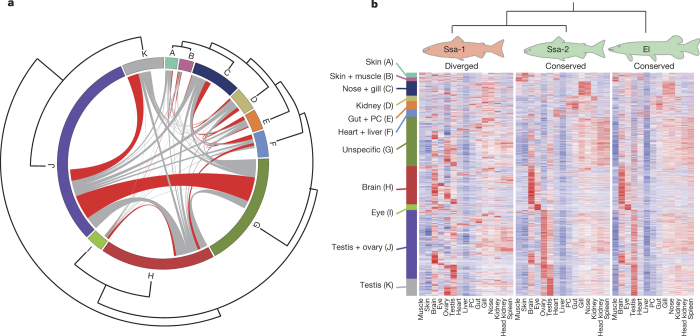
Homeologue divergence. **a**, Circos plot distribution of homeologous gene pairs and their assignment to 11 co-expression clusters based on 15 different tissues. Lines connect Ss4R pairs that belong to different co-expression clusters. For visualization purposes, we sorted the Ss4R pairs according to type of co-expression divergence. Red lines signify significant resampling tests (*P* < 0.05) for enrichment of homeologue divergence between two specific co-expression clusters. **b**, Heatmap of 2,272 triplets (two salmon homeologues and a pike orthologue), in which one of the Atlantic salmon homeologues has diverged in gene expression regulation. PowerPoint slide

**Extended Data Figure 4:**
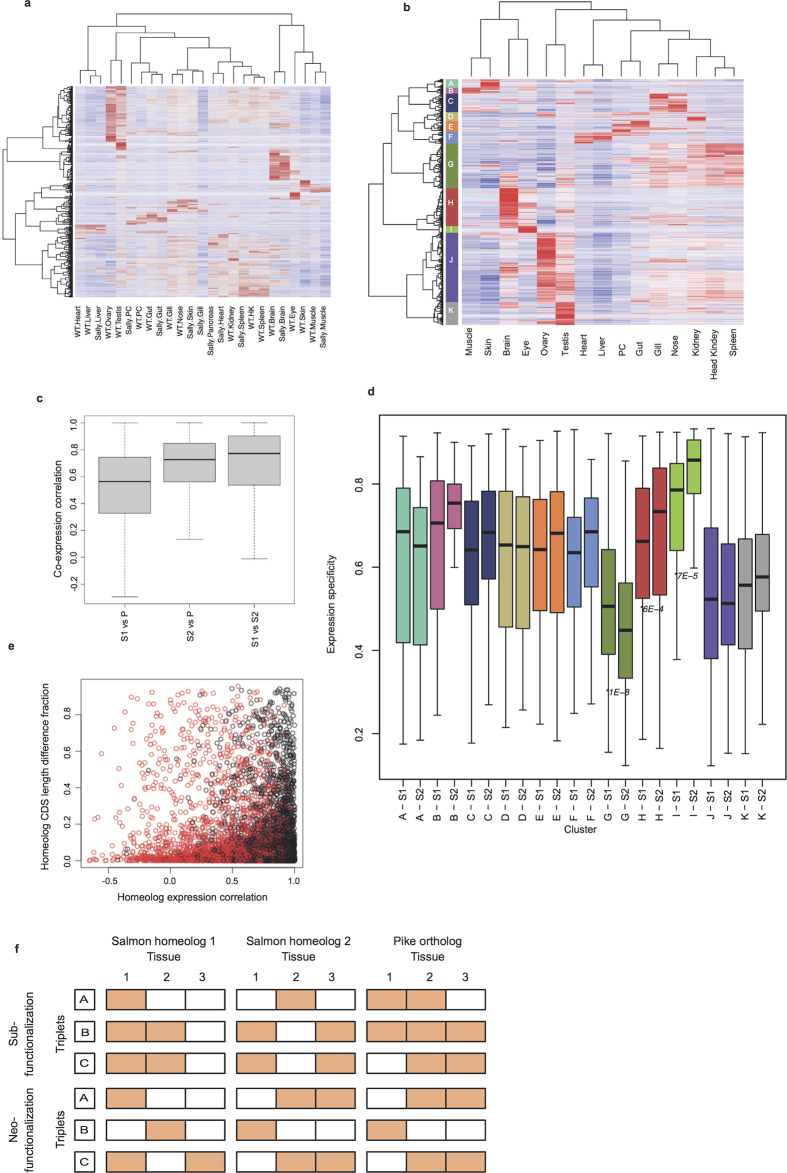
Tissue gene expression regulation. **a**, Hierarchical clustering of tissue gene expression in adult salmon from fresh water. WT = expression data from normal diploid Atlantic salmon. Sally = expression data from the double haploid fish used for reference genome sequencing. **b**, Classification of 11 co-expression clusters. Gene expression are from 15 tissues from a diploid adult Atlantic salmon from freshwater. Co-expression clusters are either associated with expression patterns from a single tissue or multiple tissues with similar physiological functions. Co-expression clusters A–K are named accordingly after the tissue(s) that contributes the most to its characteristic expression regulation profile: skin; skin and muscle; nose and gill; kidney; gut and pyloric ceca; heart and liver; unspecific; brain; eye; testis and ovary; testis. **c**, Gene expression correlation between salmon Ss4R homeologues and Northern pike orthologues. P = pike, S1 = salmon homeologue with lowest tissue expression correlation with pike, S2 = salmon homeologue with highest tissue expression correlation to. **d**, Tissue expression specificity. Tissue expression specificity of Ss4R homeologues with novel gene regulation (S1) and conserved gene regulation (S2) compared to pike. Gene co-expression clusters are denoted A–K (see description in figure legend for **b**). Significantly different tissue specificity between diverged (S1) and conserved (S2) homeologues are indicated with a *P* value in the figure. **e**, Relationship between CDS-length difference and Ss4R expression regulation divergence. CDS length divergence are calculated as a fraction of the longest CDS in each Ss4R pair. Red colour represents homeologue pairs that are in different co-expression clusters (see above sections **a** and **b** for details). **f**, Illustration of sub- and neofunctionalization as defined by the analyses of ‘on’ and ‘off’ expression patterns. Red colour indicates a gene being ‘on’ in one tissue compared to its Ss4R duplicate and the assumed ancestral state of the diploid pike outgroup.

Two major mechanisms by which a pair of duplicates can escape the fate of nonfunctionalization are subfunctionalization (partitioning of ancestral gene functions)^30^ and neofunctionalization (assigning a novel function to one of the duplicates)^31^. To assess the relative importance of these two mechanisms we analysed gene expression divergence of Ss4R duplicates across 15 tissues (Extended Data Fig. 4a, b; Supplementary Information section 7). Forty-five per cent (3,991/8,954) of well-defined expressed Ss4R pairs showed signs of diverged expression by being located in different co-expression clusters (Fig. 4a). Diverged homeologues tended to belong to closely related but still different co-expression clusters (Fig. 4a and Extended Data Fig. 4d).

Although these results suggest that functional divergence is common among Ss4R duplicates, information about ancestral state is critical for the classification of this divergence into sub- and neofunctionalization. We therefore used comparable expression data across 13 common tissues from diploid Northern pike^23^ as a proxy for the ancestral state of Ss4R duplicates. We identified 8,102 orthologous gene triplets (that is, two Ss4R copies and their putative pike orthologue) and in 42% of the triplets both Ss4R duplicates showed conserved co-expression profile with the pike orthologue (Pearson correlation >0.6, *P* < 0.03). This indicates strong purifying selection pressure on gene regulation across more than 100 Myr and adds credibility to the use of Northern pike for assessing ancestral gene regulation. In 28% of the triplets, one Ss4R duplicate had a conserved co-expression pattern with pike and the other belonged to a different co-expression cluster (Fig. 4b), indicative of regulatory neofunctionalization.

Although we observed cases of putative pseudogenization in Ss4R duplicates displaying a low correlation in expression regulation in combination with large coding sequence length difference, most Ss4R duplicates had similar lengths regardless of their expression similarity (Extended Data Fig. 4e), suggesting that neutral evolution can only marginally explain this regulatory divergence.

We identified 1,084 triplets where the salmon duplicates belonged to different expression clusters and had expression profiles significantly different from pike (Pearson correlation <0.55, *P* > 0.05), pointing to possible subfunctionalization. In this group we found, somewhat surprisingly, only 23 clear examples of subfunctionalization where the sum of the expression patterns of salmon homeologues correlated significantly with assumed ancestral state. However, this cluster-based analysis neglects subtler within-cluster subfunctionalization cases, as well as those involving acquisition of novel functions after subfunctionalization. To account for this, we applied an ‘on–off’ classification method (Extended Data Fig. 4f and Supplementary Information section 7.2) that increased the estimate to 167 cases; a figure that is still dwarfed by the estimated number of neofunctionalization cases (3,028) (Supplementary Information section 7.2).

Purifying selection on dosage sensitive interactions with other duplicated genes is thought to be an important mechanism for intermediate duplicate retention after WGDs^15^, before neo-, sub- and nonfunctionalization determine the ultimate fate of the duplicates^32^. In line with this, we observed an overrepresentation of GO terms associated with signal transduction, protein complex formation and transcription among the duplicated genes with conserved regulation (Supplementary Information section 7.3 and Supplementary Table 16). However, as a diversity of GO terms not focal to the dosage balance hypothesis (Supplementary Table 16) are also overrepresented among Ss4R duplicates with conserved regulation, it is not justified to conclude that dosage balance is the sole intermediate retention mechanism. Furthermore, analyses of retention patterns after Ts3R and Ss4R suggest independent retention probability and a very weak effect of preferential co-retention of known protein interacting partners (*P* < 0.001) for both the Ts3R and Ss4R duplication events (Extended Data Fig. 3 and Supplementary Information section 8).

Taken together, >60% of the homeologue pairs show signatures of tissue-dependent regulatory divergence at the whole gene or exon-level (Supplementary Information section 7.2). The predominance of cases where only one copy has changed its regulation compared to the assumed ancestral state indicates that regulatory subfunctionalization has not been a dominant duplicate retention mechanism post Ss4R, unless it was followed by subsequent neofunctionalization, which has been suggested as a common process^33,34^. However, our subfunctionalization estimates together with the high frequency of triplets where one salmon homeologue had a conserved co-expression pattern with pike while its duplicate did not (Fig. 4b), are not consistent with the generality of this latter scenario.

## A reference genome for salmonids

Conservation of synteny between salmonids^22,35^ suggests that information from one high-quality salmonid genome can be used to improve genome sequence assemblies of other salmonids. To test the feasibility of such a comparative genomics approach, we used the Atlantic salmon assembly to construct chromosome sequences for the non-chromosome anchored rainbow trout genome sequence^13^. We were able to map 99.5% of rainbow trout scaffolds >100 kilobases (kb) (total 1.22 Gb) to the Atlantic salmon chromosome sequences (Supplementary Information section 1.5).

Using the Atlantic salmon chromosome sequences together with a dense linkage map for rainbow trout constructed from a 57K single nucleotide polymorphisms (SNP) array, we were able to anchor, orient and concatenate 11,335 rainbow trout scaffolds (scfN50 = 940 kb, from ref. 13) into 29 rainbow trout chromosome sequences (Supplementary Information section 9). This was done by first using the rainbow trout linkage map to determine the proximate order of 2,439 trout scaffolds containing SNPs, which we found to be sufficient for determining conserved blocks. Then we used comparative information from Atlantic salmon to incorporate scaffolds without SNP information, and fine-tune the order and orientation of all 11,335 trout scaffolds into chromosome sequences. Even though the rainbow trout linkage map contains more markers than most other salmonids (for example, ref. 22), this high number of properly placed scaffolds would not be achievable without the Atlantic salmon information.

Alignment of these rainbow trout chromosomes (representing 1.37 Gb of sequence) with the Atlantic salmon genome revealed conservation of very large syntenic blocks, in many cases corresponding to whole chromosome arms in rainbow trout (Extended Data Fig. 1). This analysis supports previous results^35^ suggesting conservation of 50 syntenic regions representing the karyotype of 50 acrocentric chromosomes in the common ancestor of salmonids^36^. Our analysis documents that these syntenic regions typically represent blocks with no rearrangements for 38 regions and with only one or two inversions or translocations among the remaining parts.

## Implications

The conservation of large collinear blocks between *Salmo* and *Oncorhynchus* strongly suggests that the Atlantic salmon genome information will facilitate exploitation of genomic information in a wide range of ecological, evolutionary, conservation and production biology settings within salmonids. Moreover, the availability of a high-quality assembly and annotation of the Atlantic salmon genome provides novel insights into vertebrate post-WGD evolution that may contribute to a more thorough understanding of the underlying mechanisms as well as the long-term importance of WGD for adaptation.

## Methods

### Data reporting

No statistical methods were used to predetermine sample size. The investigators were not blinded to allocation during experiments and outcome assessment.

### Genome sequencing and assembly

DNA from a single double-haploid female from the AquaGen strain, produced by mitotic androgenesis, served as the template for sequencing using Sanger and next generation sequencing technologies (Supplementary Table 1). Various assemblies were generated using different combinations of software and subsets of data (Supplementary Table 2). The foundation of the chosen assembly was generated from Sanger (~4×) and Illumina (~202×) data assembled using the MaSuRCA (v2.0.3) assembler^37^. The assembly was reconciled and gap-filled using information from preliminary assemblies (Supplementary Information section 1.3). Genetic linkage information describing 565,877 SNPs was used to both confirm and correct scaffolds and, when supported by information from other assemblies, was used to join scaffolds within linkage groups. Subsequently, linkage analysis using CRIMAP^38^ and a subset of SNP sequence tags (27,221) were used to order, orient and concatenate scaffolds into 29 single-chromosome sequences. Nomenclature for Atlantic salmon chromosomes is based on ref. 35.

### Gene annotation

Gene structures were determined by combining data from full-length cDNA sequences^39^, EST databases^39,40,41^, and RNA-seq data from 15 tissues (Supplementary Table 9). RNA-seq reads were trimmed using Trimmomatic (v0.32 (ref. 42)) and mapped to the reference genome sequence using STAR (v2.3.1z12 (ref. 43)), and all publicly available mRNAs and ESTs were mapped using GMAP^44^. Gene structures were predicted with CUFFLINKS^45^. Open reading frame (ORF) predictions were carried out using TransDecoder^46^. Gene models without homology match to either PFAM, stickleback or zebrafish were discarded. Functional annotation was done with Blast2GO^47^ against the SwissProt database. Transposable element related ORFs were identified with BLAST searches against the annotated transposable element sequences and queries in the functional annotation gene names for transposable element related terms (that is, retrotransposon, transposon, transposable, transposase, reverse transcriptase, gag, bpol). Putative expressed and silenced Ss4R homeologues were identified using a combination of homology searches with BLAST and GenomeThreader^48^ targeting a priori defined conserved collinear duplicated regions (*n* = 98).

### Repeat library methods

An Atlantic salmon repeat library of 2,005 elements was assembled from sequences previously reported in salmonids^13,49,50^ and the output of the *de novo* repeat-finding programs LTRharvest^51^, RepeatModeller^52^ and REPET^53^. With the exception of curated repeats previously reported by Matveev and Okada^50^ and those found in the RepBase database^49^, all preliminary sequences were validated using BLASTn^54^ to ensure that they were present at multiple locations in the genome. LTRharvest sequences were filtered based on the repeat library construction procedure outlined in the MAKER documentation^55^. Using BLASTn, sequences from other *de novo* sources and the rainbow trout repeat library were flagged as potentially chimaeric if they did not generate at least three high-scoring segment pairs (HSPs) covering at least 80% of their length in the Atlantic salmon genome. Any distinct highly repetitive region within such sequences was extracted and retained while other portions were discarded. All libraries were merged and redundant sequences were removed based on the guidelines presented by Wicker *et al*.^56^ and the MAKER documentation. Sequences in the combined library were annotated, and non-transposable element host genes were removed based on their similarity to well-characterized sequences in annotation databases^49,57^, the presence of structural motifs and manual examination.

To estimate the historical activity of Tc1-*mariner* transposable elements, up to 100 randomly selected full-length genomic copies from each of 40 Tc1-*mariner* families were extracted and aligned using MUSCLE^58^. All families were confirmed to be phylogenetically distinct from each other and possessed a star-like neighbour-joining tree topology characteristic of Tc1-*mariner* activity^59^. The distribution of pairwise per cent similarity, a proxy for time, between members of a family was used to analyse the temporal dynamics of transposable element activity.

### Identification of homeologous blocks within the salmon genome

Repeat masked chromosome sequences for Atlantic salmon (see above) were aligned against each other using LASTZ^60^ to identify 98 homologous blocks originating from the Ss4R (for details see Supplementary Information section 2). Sequence similarity between homeologous sequences were determined in 1 Mb intervals by averaging local percentage of nucleotide sequence identity using high-scoring segment pair (HSP) from LASTZ alignments^60^ and presented as a Circos plot^61^ in Fig. 2.

### Sequence evolution analyses of salmon homeologues

Putative orthologue sequence sets were collated with Best Reciprocal Blast (BRB) protein matches. For salmonid species the top-two BRB-hits were assigned to putative orthologue groups. Multiple codon sequence alignments were constructed using MAFFT^62^ and quality trimmed with Guidance in an iterative framework where sequences were re-aligned after identification of poorly aligned codons.

Maximum likelihood (ML) gene trees were calculated by the R-package Phangorn^63^ using codon alignments, the GTR+G+I model, and 100 bootstrap replicates. Branch specific GTR+G+I substitution rates were estimated functions from the R-package ape^64^, while branch specific synonymous (dS) and non-synonymous (dN) substitution rates were estimated with non-negative least squares regression in the Phangorn R package^63^ using pairwise dN and dS distance matrixes from codeml^65^ and the ML gene tree topologies as input.

Branch-site specific test for positive selection was carried out by a likelihood-ratio test on the ML-likelihood estimates for sequence evolution under different models in codeml. The smallest likelihood estimate from four omega starting values (0.5, 1, 1.5, and 2) was used in the likelihood ratio test (LRT). False discovery rate adjustments of p-values were done with the p.adjust function in R.

### Gene tree dating

BEAST^66^ was used to calibrate gene trees using a HKY+G substitution model, uncorrelated lognormal clock, and yule tree prior. The BEAST analyses were exclusively based on codon alignments that produced a ML-gene tree topology containing two Ss4R homeologues in both *Salmo* and *Oncorhynchus*, and where rediploidization had occurred before the *Salmo–Oncorhynchus* divergence. No priors on tree topology were specified and a single secondary calibration of 127 Myr (confidence interval 12.5 Myr) on the most recent common ancestor of Salmoniformes + Esociformes was used^7,8^. All Markov chain Monte Carlo (MCMC) analyses were run for 10 million generations with sampling every, 1000 generations. Tracer v1.6 (available from http://beast.bio.ed.ac.uk/Tracer) was used to inspect effective sample sizes (ESS) of tree parameters. Fifty per cent consensus topologies were constructed based on 100 randomly sampled tree topologies from the last 1,000 MCMC-samples. Age of *Salmo–Oncorhynchus* divergence was estimated as the median of two nodes per tree.

### Transcriptome analysis

A gene was classified as ‘expressed’ if the FPKM value of at least one tissue was above 1.0, and values were transformed to log_2_ (FPKM+1) values for consecutive analysis. Samples and genes were clustered using Pearson correlation and Ward’s method in the R function hclust^67^, and visualized as heatmaps using the R function heatmap.2 (gplots library). Genes were scaled individually in the heatmaps.

Clusters with a significant number of shared homeologue-pairs were identified by simulation (10,000 randomizations). A salmon gene (or exon) was classified as conserved if the Pearson correlation to the pike orthologue was above 0.6 (*P* = 0.03) across the 13 common tissues, and diverged if the correlation was below 0.55 (*P* > 0.05). A salmon homeologue-pair was classified as neofunctionalized if at least one salmon gene was conserved and the two salmon genes were in different clusters, and as subfunctionalized if both salmon genes were diverged and in different clusters, but their summed expression was conserved.

Expression specificity was computed as one minus the sum, over all samples, of the gene’s expression in that sample divided by the maximum expression in any sample. Significant difference in specificity between clusters was computed using the Wilcoxon test.

### Duplicate retention

Existing gene families for all teleost species were downloaded from Ensembl Compara 79 (ref. 27). Genomes for *Salmo salar*, *Esox lucius*, and *Oncorhynchus mykiss* were added to these gene families or used to create new gene families with BLAST to determine homologous relationships (*e*-value >1*e*-10 and %id>50)). Multiple sequence alignments of extended gene families with *Lepisosteus oculatus* as an outgroup were produced using MAFFT^62^ (command line option –auto) and gene trees were built with PhyML 3.4 (ref. 68) using the JTT+G substitution model. Using the NCBI teleost species tree, Softparsmap^69^ was used to identify duplication and speciation event in trees. This resulted in 12,388 gene families with a speciation root node, encompassing 26,325 salmon genes.

The constructed gene trees were then assessed for duplicate retention for the Ts3R, Ss4R, small scale salmon specific duplications (SSD) following the Ss4R event, and duplications occurring between the Ts3R and Ss4R. Duplicate retention was counted by examining the conditional percentages of genes that were retained from the Ss4R following the Ts3R, and from the Ss4R to small-scale duplications on the salmon lineage. The duplication lineage for each gene was counted, ensuring that each lineage accounted for the retention or loss of a duplicate, with the expectation that each Ts3R duplication should give rise to two Ss4R, and every Ss4R should lead to two small scale duplications. Post3R–preSs4R SSDs also share an expectation of having resulted in two Ss4R duplications. Where nodes could be assigned as being either Ss4R or SSD, the chromosomal locations of the genes were used to differentiate between the ambiguous nodes. Such ambiguous nodes were determined to be SSDs if the duplicate salmon genes resided on the same chromosome; otherwise it was classified as being Ss4R. Since only a single Ss4R duplication occurred along a lineage, if two ambiguous nodes were found that could be classified as Ss4R along the same lineage, one was classified as being Ss4R and the rest were classified as being SSD, with the oldest duplication being the Ss4R, an assumption that did not affect the trends in the data. Although most gene tree topologies were consistent with the teleost species tree, some gene trees showed large deviations from the accepted species tree. These trees may have been influenced by phylogenetic error which could cause spurious duplication counts and cause an overestimation of the number of duplication events within a gene family. Conditional probabilities were then calculated to determine the fraction of retained gene duplicates following each of the WGDs, given the opportunity for retention.

To assess if duplicate retention was impacted by protein–protein interactions, known protein–protein interactions were downloaded from the STRING database^70^. BLAST against *Danio rerio* was performed and putative STRING interactions in salmon were determined. Only interactions labelled ‘binding’ were kept, which are putative physical protein–protein interactions based on various forms of evidence. Patterns of co-retention following Ts3R, Ss4R, and SSD were then examined among STRING binding partners using the phylogenetic trees described above with custom perl scripts.

Statistical tests of significance were performed to determine if duplication counts were significantly different from each other. The duplication process was represented by a binomial distribution where each duplication could have either been retained or not. A two-proportion pooled *z*-test was performed to calculate two-sided *P* values at the Bonferroni corrected α-level (0.001/7). To further explore if results were significant with a marginal effect level change or being overly influenced by large sample sizes, an odds ratio and relative risk analysis was performed for each group and two-sided *P* values were calculated. All tests showed extremely low *P* values indicating that the groups were significantly different from one another^71^. Effect sizes were considered as the fractional change in mean values.

All scripts used in this analysis are freely available on the Liberles Group website at Temple University (USA) at https://liberles.cst.temple.edu/public/Salmon_Genome_Project/.

### Use of salmon assembly to improve rainbow trout genome sequence

Salmon chromosome sequences were repeat masked using a salmon repeat database and RepeatMasker v4.0.3 (ref. 72) and aligned against rainbow trout scaffolds^13^ using MegaBLAST^73^. Rainbow trout scaffolds mapping to multiple salmon chromosomes were broken when supported by information from a rainbow trout linkage map containing 31,390 SNPs constructed in a family material of 2,464 individuals using Lep-MAP^74^. The relative positions of trout scaffolds within the salmon genome were used, together with trout linkage maps, to position, orient and concatenate 11,335 rainbow trout scaffolds into 29 single chromosome sequences (1.37 Gb). Nomenclature for rainbow trout chromosomes is based on ref. 35. Conserved syntenic blocks between rainbow trout and Atlantic salmon were determined by aligning chromosome sequences for the two species against each other using LASTZ^60^.

## Supplementary information

Supplementary Information This file contains Supplementary Text and Data, Supplementary Tables 1-3, 5, 7-8, 10-17 and Supplementary References – see contents page for details. (PDF 2077 kb)

Supplementary Data This file contains Supplementary Table 4. (XLSX 12 kb)

Supplementary Data This file contains Supplementary Table 6. (XLSX 15 kb)

Supplementary Data This file contains Supplementary Table 9. (XLSX 12 kb)

## Figures and Tables

**Extended Data Figure 5:**
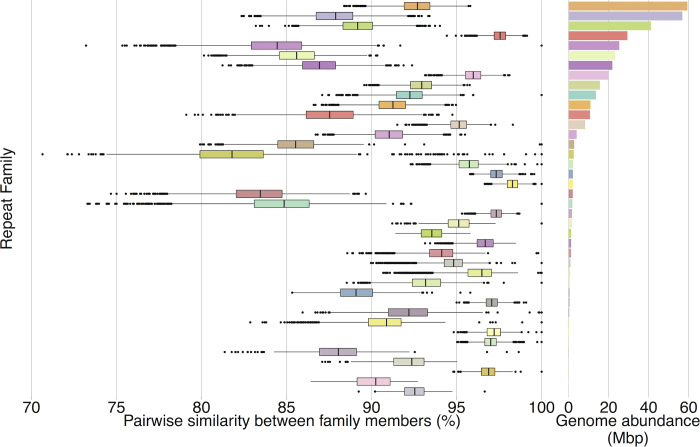
Historical activity of 40 Tc1-*mariner* transposable elements and their abundance in the Atlantic salmon genome. Families with increased pairwise similarity between members have experienced less neutral sequence divergence since they were rendered inactive and reflect more recent additions to the genome.

## Data Availability

NCBI Reference Sequence
GCA_000233375.4 Sequence Read Archive
PRJNA260929
PRJNA72713 Sequence information was deposited at GenBank under accession code GCA_000233375.4 and at the NCBI Sequence Read Archive (SRA): PRJNA72713 and PRJNA260929.

## PowerPoint slides

PowerPoint slide for Fig. 1
PowerPoint slide for Fig. 2
PowerPoint slide for Fig. 3
PowerPoint slide for Fig. 4
