# Supplementary material for: The Atlantic salmon genome provides insights into rediploidization
Source: Nature. 2016 Apr 18;533(7602):200–5. doi: 10.1038/nature17164 (PMC8127823; doi:10.1038/nature17164)
Supplement: Supplementary Information — This file contains Supplementary Text and Data, Supplementary Tables 1-3, 5, 7-8, 10-17 and Supplementary References – see contents page for details. (PDF 2077 kb) [file 41586_2016_BFnature17164_MOESM9_ESM.pdf]

doi:10.1038/nature17164

1. Genome sequencing and assembly
   1.1. Sequencing
   1.2. *de novo* assembly
   1.3. Assembly refinement
   1.4. Atlantic salmon chromosome sequences
   1.5. Validation of assembly quality
2. Resolving homeologous regions in the salmon genome
3. Salmon repeats
   3.1. Preliminary repeat libraries
   3.3. Removing redundant, low-repetition and chimeric sequences
   3.4. Host gene detection and removal
   3.5. Repeat classification
   3.6. Tc1-Mariner activity history
4. Gene Annotation
   4.1. Identification of gene structures
   4.2. Classifying Ss4R gene duplicates
5. Evolution and retention of Ss4R homeologs
   5.1. Estimation of ortholog/homeolog gene trees
   5.2. Comparative gene tree content analyses
   5.4 Analyses of sequence evolution
   5.5. Test for positive selection
6. BEAST dating of homeolog rediploidization times
   6.1 Gene tree calibration
7. Analyses of transcriptomics
   7.1. Tissue specific Expression in salmon
   7.2. Evolution of homeolog gene regulation
   7.3. GO analyses of Ss4R with conserved and diverged gene regulation
8. Ts3R and Ss4R duplicate retention
   8.1. Gene tree based duplicate retention analyses
9. Reference genome for salmonids

References

1. Genome sequencing and assembly

1.1. Sequencing.

DNA from a single double-haploid female from the AquaGen aquaculture strain, produced by mitotic androgenesis[1] served as template for all sequencing (BioSample: SAMN02749551; Sample name: Sally). Eggs were irradiated for 60 minutes with a Cobalt 60 source at a dose ranging from 0.18 – 0.60 kGy. Following fertilization eggs were heat shocked at 31.5 ºC for five minutes and allowed to develop at 7 °C.

Sequence data is summarized in Supplementary Table 1. The total sequence data covers the hypothetical 3 Gb genome 222 times. Sequence reads were submitted to the NCBI SRA[2] under BioProject PRJNA72713, Study SRP011583.

Sanger. Whole genome shotgun libraries were prepared using standard methods and sequenced on ABI 3730xl instruments. Insert sizes averaged approximately 3 kb for plasmid, 38 kb for fosmid, 145 kb for BAC clones. Sanger reads were processed with the Peak Trace base caller (Nucleics).

Illumina. Illumina paired-end (PE) libraries were constructed with insert sizes ranging from 180 bp to 600 bp. Illumina mate-pair (MP) libraries were constructed targeting insert sizes of 3 kb, 8 kb, and 20 kb. Eleven attempts to generate tight, large-insert libraries were largely unsuccessful and bioinformatic analysis indicated that libraries contained a predominance of 3 kb inserts. Illumina libraries were sequenced on Illumina GAIIx, HiSeq 2000, and MiSeq machines.

PacBio. High molecular weight DNA agarose blood plugs were made according to the supplementary protocol of[3] and stored in 0.5 M ethylenediaminetetraacetic acid (EDTA). DNA was dissolved overnight in 1 ml of Tris-EDTA buffer, purified with GELase (EpiCentre) the remove additional agarose, and up-concentrated using Genomic-tip 100/G (Qaigen) before being used for PacBio library preparation. SMRTbell libraries were prepared with insert sizes in the 10 to 20 kb range. Blue Pippin size selection was applied to the final library. The libraries were sequenced on PacBio RS machines, versions I and II, using C2 and C2XL chemistries, and processed with instrument software versions 1.3, 1.4, and 2.0. Library construction and sequencing was performed by the following centers: Beckman Coulter (USA), University of Victoria (Canada), the J. Craig Venter Institute (USA), Norwegian Sequencing Centre, University of Oslo (Norway), Pacific Biosciences, Menlo Park (USA), and Case Western Reserve University (USA). Separately, individual packs were Illumina paired-end sequenced by BGI-Shenzhen (China).

Supplementary Table 1. Atlantic salmon sequencing. Atlantic salmon was sequenced to over 220X read coverage using Sanger, Illumina, and PacBio platforms. The Sanger reads sequences were initially called using KB base caller from Applied Biosystems (Thermo Fisher, USA) and subsequently re-processed using Peak Trace base caller from Nucleics [https://www.nucleics.com/peaktraces/PeakTrace%20Whitepaper.pdf]. Sanger insert sizes were approximately 3 kb for plasmids, 35 kb for fosmids, and 120 kb for BACs. The Illumina GAIIx read lengths varied by run from 114 to 151 bp. The Illumina MP insert size range was 3 kb and larger with a 3 kb mode. Coverage figures assume a 3 Gb genome size.

Platform	Library type	Read type	Reads	Bases	Coverage	Avg Read Len
Sanger	plasmid	paired	12 085 472	11 641 917 181	3.9	963
Sanger	fosmid	paired	3 004 571	2 368 551 451	0.8	788
Sanger	BAC end	paired	280 410	258 079 410	0.1	920
GAIIx	PE	2x150	1 433 383 064	200 496 215 400	66.8	140
HiSeq	PE	2x100	993 419 338	99 341 933 800	33.1	100
MiSeq	PE	2x250	109 246 228	27 420 803 228	9.1	251
GAIIx	MP	2x150	612 862 444	92 542 229 044	30.8	151
HiSeq	MP	2x100	1 734 152 804	175 149 433 204	58.4	101
PacBio	C2XL	unpaired	19 815 718	57 589 215 962	19.2	2 906
**Total**			4 918 250 049	666 808 378 680	222.3	136

1.2. Preliminary assembly.

AGKD. This initial assembly (GCA_000233375.1) was released to GenBank in 2011. The input Sanger reads had not yet been re-processed using PeakTrace (Nucleics) and were the product of KB base caller. The available Illumina data consisted of two full runs of GAIIx PE sequencing. Arachne[4] was used to assemble the Sanger data, ABySS the Illumina[5], and PCAP[6] was used to combine the outputs of both assemblers. The assembly consists of contigs without scaffolds. This assembly has labels GCA_000233375.1, ASM23337v1, and ICSASG_v1.

AP1. ALLPATHS-LG[7] was used to assemble the Illumina HiSeq 2x100 overlapping PE reads, the Illumina GAIIx data from six 3 kb-8 kb insert libraries, and the Sanger fosmid mates. ALLPATHS (CacheReadsMerge module) reported inputs after filtering of 50X fragment reads (Illumina PE) with 86% pairs overlapping, 50X long jump (Illumina 3 kb-8 kb MP), and 3.4X long jump (Illumina 20 kb MP and Sanger fosmid). Based on its own reporting: ALLPATHS used 914 GB RAM, 5.9 TB disk, and 3.3K CPU hr; it had insufficient data to re-estimate long jump insert sizes but it re-estimated all other jump inserts to between 2,497 and 3,687 bp; it estimated a genome size of 2,787,646,091 based on its K-mer analysis; it generated 354,746 contigs with N50 4.8 kb based on 1,176,567,533 bases in contigs; and it generated 47,940 scaffolds with N50 297 kb based on 1,873,720,400 scaffold span.

AP2. ALLPATHS-LG was run on all Illumina PE and MP data plus Sanger fosmid mates and Sanger BAC ends. The ALLPATHS CacheReadsMerge module reported inputs after filtering of 26X fragment reads (Illumina PE) with 72% pairs overlapping, 50X long jump (Illumina 3 kb-8 kb MP), and 3.4X long jump (Illumina 20 kb MP, Sanger fosmid, and Sanger BAC-ends). ALLPATHS estimated a genome size of 2,328,417,603 based on its K-mer analysis; it generated 303,224 contigs with N50 9.6 kb based on 1,699,656,308 bases in contigs; and it generated 24,971 scaffolds with N50 1162 kb based on 2,120,995,472 scaffold span.

SSEP1, CA7. Celera assembler[8,9] was run on several combinations of reads. The SSEP1 assembly applied CA6 to Sanger and Illumina GAIIX data after Celera Assembler was modified to handle upwards of 1G reads. The assembly pipeline included the merTrim step to error-correct the Illumina base calls and the OBT step to trim the Sanger. The CA7 assembly applied CA7 to the same data and included interventions to address genome repetitiveness. Celera Assembler builds contigs and scaffolds from its "unitig" database called tigStore. For this assembly, the tigStore constructed through command-line concatenation of three preliminary stores. The first store was constructed by mapping all reads to a database of salmon repeats. The second store was constructed by *de novo* assembly of unmapped reads. The third store was constructed by extracting reads from small (3 reads or fewer) unitigs in the second store and re-assembling them together with no limit on K-mer frequencies used to seed overlaps.

BBB. An approximate tiling of BACs were sequenced and assembled by BGI-Shenzhen (China) using the SOAPdenovo2 assembler.

PB1, PB2. Two strategies were employed to overcome high base call error in individual PacBio reads. In the first strategy, PacBio reads were pre-processed with the pb2ca pre-assembly base call correction software[10] using Illumina paired end read evidence. Sanger and corrected PacBio were trimmed separately with the Celera Assembler version 8 (CA8) overlap-based trimming module (OBT) and CA8 ran on the combined results. Despite several attempts, these assemblies yielded small contigs and failed to produce scaffolds in reasonable time. In the second strategy, the PacBio reads were assembled without base call correction. CA8 had been modified to recognize and trim subreads in PacBio data, to find overlaps with up to 40% alignment error tolerance, and to snip apparent repeat structures from the overlap graph made of PacBio reads. Sanger and PacBio reads were separately trimmed with CA8 OBT and assembled with CA8 to yield the PB1 assembly. Analysis of PB1 revealed several redundant unitig placements in scaffolds. This led to the detection and correction of a software problem in CA8. The PB2 assembly was generated by re-starting the PB1 assembly at the scaffold stage using updated CA8 software.

1.3. The final assembly series

An early assembly named ASM3.0 underwent progressive refinement to produce the ASM3.6 final assembly.

ASM2.0, ASM3.0. The raw Sanger and Illumina data were assembled with MaSuRCA[11]. The Illumina data were analyzed for K-mer content with the Jellyfish K-mer counter[12]. A de Bruijn graph was constructed from Illumina lanes with a low portion of low-frequency K-mers. So-called super-reads were extracted from the de Bruijn graph to provide approximately 3X uniform coverage of the genome. Additional super reads were extracted based on the input Illumina MP. The Sanger reads and super reads were assembled with MaSuRCA's version of Celera Assembler, which had been modified to transfer input read coverage information from the super reads to the CA unitigs. MaSuRCA generated ASM3.0 using Sanger, GAIIx, HiSeq, and MiSeq reads. A similar process generated ASM2.0 from Sanger and Illumina GAIIx using an early version of MasuRCA named MSR-CA.

ASM2.1, ASM3.1. The MaSuRCA gap fill post-process was applied to two assemblies. The process performs local assembly at intra-scaffold gaps. It incorporates the neighbor contig sequences, the gap size estimates, and Kmers from previously unincorporated sequence. A similar process generated ASM2.1 using MSR-CA.

ASM3.2. The ASM3.2 assembly was derived from integration of linkage data with ASM3.1 by methods outlined below for ASM3.6.

ASM3.4. The ASM3.4 assembly was derived from ASM3.2 using sequences from three prior assemblies: ASM2.1, AP1, and PB1. The scripted process, similar to Assembly Reconciliation[13], joined two scaffolds of ASM3.2 into one scaffold if any one of the prior assemblies supported an end-to-end join. Reconciliation was followed by a final round of gap fill using pseudo-reads extracted from the contigs of three prior assemblies: ASM2.1, AP1, and PB1.

ASM3.6. The ASM3.6 assembly was derived from ASM3.4 through integration of linkage data. A dense SNP-array containing approx. 930K SNPs developed for Atlantic salmon was genotyped in a family material of 840 samples from a commercial breeding population (AquaGen, Norway). In total 565,877 SNPs passed quality control and displayed minor allele frequencies higher than 0.025 in the family material. A linkage analysis pipeline was developed and used to integrate these SNPs with an anchor map containing 5,650 SNPs previously assigned with high confidence to the 29 salmon chromosomes[14]. Most likely position of the salmonXHD SNPs in relation to the anchor map was determined by two-point linkage analysis. Sequence flanking each marker was used to precisely position all SNPs to contigs and scaffolds from the ASM3.4 assembly using megablast and thereby associate sequence with linkage groups. This revealed a large number of chimeric scaffolds containing SNPs from different linkage groups that were then selectively 'broken' between, apparently, incorrectly linked contigs if the conflicting assignments were either; 1) supported by a large number of markers on both sides, or 2) supported by a moderate number of markers but lacking any confirmation in another assembly. After breakage of scaffolds linkage, information was used together with the PB2 assembly to join both broken and unbroken scaffolds

within chromosomes to produce the ASM3.6 assembly. A final round of scaffolding of the ASM3.6 assembly was performed using SSPACE Basic v2.0 (parameters: -k 1)[15] on Sanger fosmid mates and Sanger BAC ends aligned using BLASR v1.3.1[16] (parameters: -minPctIdentity 98 -minMatch 500) with only uniquely mapped sequences as input to SSPACE.

1.3. Assembly results.

Assemblies were compared by scaffold N50 and contig N50 computed on a 3 Gb genome size for N50 comparability between assemblies; see Supplementary Table 2. Each N50 statistic indicates that 50% of the assumed genome size was assembled in contigs (or scaffolds) of that size or greater. The ASM3.0 assembly (MaSuRCA applied to Sanger and Illumina) generated the largest scaffold N50 by a wide margin. The PB1 and PB2 assemblies generated the largest contig N50 sizes (Celera Assembler applied to Sanger and uncorrected PacBio) though though relatively small scaffolds and higher-than-expected total contig bases. The ASM3.0 assembly was selected for refinement by reconciliation with other assemblies and the genetic map, as described above. The iterative refinement process started with ASM3.0 and finished with ASM3.6; see Supplementary Table 3. Refinement reduced the number of contigs and scaffolds by 23% while increasing the contig N50 by 45% and the scaffold N50 by 32%. Almost 250K scaffolds were filtered but these tended to be small (1 kb N50). The number of bases decreased by 3% overall due to filtering as well as joins that involved sequence alignment. The final assembly submitted to GenBank (labels GCA_000233375.4 and ICSASG_v2) is described in Supplementary Table 4. This assembly has a 57.6 kb contig N50. About two thirds of the assembly is mapped to chromosomes and has a 2.97 Mb scaffold N50.

Supplementary Table 2. Assembly methods and metrics. The Atlantic salmon genome assembly and selected pre-cursors. The table includes each assembly's contig N50, scaffold N50, and sum of contig bases. The N statistics assume a 3 Gb genome. Asterisks indicate the outputs that contribute directly to the final assembly. Inputs reflect the total sequence available before trimming or filtering.

ID	Inputs	Methods	ctg bases	N50 ctg	N50 scf	
**AGKD**	Sanger 3X, Illumina 38X (GAIIX)	Hybrid assembly. Arachne on Sanger. ABySS on Illumina. Combine with PCAP.	1 147 060 872	0	n/a	
**SSEP1**	Sanger 4X, Illumina 136X (GAIIX)	*De novo* assembly. Celera Assembler v6.	2 785 378 257	12 367	418 862	
**CA7**	Sanger 4X, Illumina 194X (GAIIx, HiSeq)	Hybrid assembly. Celera Assembler v7 with pre-assembled repeat unitigs.	3 924 095 645	965	128 855	
**ASM2.0**	Sanger 4X, Illumina 194X (GAIIx, HiSeq)	*De novo* assembly. MSR-CA v1.8.3.	2 890 792 596	15 657	1 598 257	*
**ASM2.1**	ASM2.0 and its unscaffolded sequences	Post-processing. MSR-CA gap fill.	2 892 271 506	19 339	1 596 346	*
**AP1**	Selected Illumina PE and MP, Sanger fosmid	*De novo* assembly. ALLPATHS-LG.	1 187 383 848	0	99 863	*
**AP2**	All Illumina PE and MP, Sanger fosmid and BC	*De novo* assembly. ALLPATHS-LG.	1 716 442 768	2 445	355 572	*
**PB1**	Sanger 4X, PacBio 19X	*De novo* assembly. Celera Assembler v8.	3 913 175 288	58 013	200 778	*
**PB2**	Unitigs from PB1 assembly	Scaffolds, Celera Assembler v8 update.	3 789 212 036	52 840	285 172	
**BBB**	Illumina 200X PE (HiSeq) from individual BACs	*De novo* assembly. SOAPdenovo applied separately to each BAC.	2 972 015 295	14 461	19 619	

**ASM3.0**	Sanger 4X, Illumina 202X (GAIIx, HiSeq, MiSeq)	*De novo* assembly. MaSuRCA v2.0.3.	3 156 339 852	26 453	1 695 527	*
**ASM3.1**	ASM3.0 and its unscaffolded sequences	Post-processing. MaSuRCA v2.0.3 gap fill.	3 158 383 897	34 043	1 694 584	*
**ASM3.2**	ASM3.1, genetic linkage map	Post-processing. Break and merge scaffolds.	3 156 339 852	26 617	1 165 465	*
**ASM3.4**	ASM3.2 and its unscaffolded sequences, contigs from PB1 and AP1 and ASM2.1 assemblies	Post-processing. Reconcile. Gap fill.	3 155 507 105	37 475	1 073 743	*
**ASM3.6**	ASM3.4, genetic linkage, PB2 scaffolds	Post-processing. Break and merge scaffolds.	3 069 555 617	38 456	1 144 865	

Supplementary Table 3. Assembly refinement. The initial *de novo* assembly ASM3.0 as refined using techniques of gap fill, assembly reconciliation, and genetic linkage map integration to generate ASM3.6. Refinement reduced the number of contigs and scaffolds by 23%. The process had a positive effect on the contig size distribution but a mixed effect on scaffolds. The largest single gain in contig N50 was achieved by MaSuRCA's gap fill post-process, reaching 34 kb in ASM3.1. Refinement reduced by 3% the number of bases in contigs and the span of scaffolds. Alignment statistics reflect the projection (union) of intervals of bwa alignments measured along each assembly. The alignment statistics indicate 4% reduction in aligned bases and 19% increase in N50 size of the aligned spans; both changes are attributable to collapse of previously distinct contigs and scaffolds. Alignment by 'bwa mem –H'.

	ASM3.0	ASM3.6	Change	Change
**Contigs**				
**Count**	1 264 512	976 039	-23 %	-288 473
**Bases in**	3 156 339 852	3 069 555 617	-3 %	-86 784 235
**N10 of**	156 130	244 323	56 %	88 193
**N50 of**	26 453	38 456	45 %	12 003
**Scaffolds**				
**Count**	1 095 170	843 055	-23 %	-252 115
**Bases in**	3 503 258 281	3 395 522 151	-3 %	-107 736 130
**N10 of**	10 027 528	10 209 906	2 %	182 378
**N50 of**	1 695 527	1 144 865	-32 %	-550 662
**Scaffolds unchanged**				
**Count**	803 130	803 130		
**Bases in**	898 553 502	898 553 502		
**N10 of**	12 895	12 895		
**N50 of**	1 016	1 016		
**Scaffolds discarded**				
**Count**	248 739			
**Bases in**	103 009 354			
**N10 of**	4 190			
**N50 of**	373			
**Alignment projection**				
**Count**	924 478	910 365	-2 %	-14 113
**Bases in**	3 052 736 737	294 344 329	-4 %	-108 392 408
**N10 of**	703 405	895 412	27 %	192 007
**N50 of**	68 622	81 593	19 %	12 971

1.4. *Atlantic salmon chromosome sequences*

Linkage data was integrated with the ASM3.6 assembly to produce chromosome sequences for Atlantic salmon. To simplify the linkage analyses we selected up to 15 SNPs per scaffold based on high minor allele frequency and position within scaffold. These 27,221 highly informative SNPs were subsequently used in linkage analyses to confirm assignment of scaffolds suggested by the published anchor map[14], assign additional scaffolds to chromosomes, order and orientate scaffolds and build sequences for 29 Atlantic salmon chromosomes. Nomenclature for Atlantic salmon chromosomes follows[17]. As a result 9,447 scaffolds (scfN50 = 2.97 Mb) representing 2.24 Gb of sequence were used to produce 29 single chromosome sequences, available at NCBI GenBank with assembly accession number GCA_000233375.4 (Supplementary Table 4).

1.5. *Validation of assembly quality*

To examine the completeness of the chromosome sequences we mapped the sequences to 498,245 publically available ESTs[18-20] and rainbow trout scaffolds[21] being larger than 100 Kb (2429 scaffolds) using megablast[22]. Prior to the alignment salmon chromosome sequences were repeat masked using a salmon repeat database (ssal_repeats_v2.0) and RepeatMasker v4.0.3[23]. After filtering for identity (>95%) and coverage (>80%), more than 95% of ESTs were position to chromosomes. A high level of completeness was also found for the alignment of rainbow trout scaffolds with more than 99.5% of scaffolds (adding up to 1.22 Gb) aligning confidently (>90% ident, >600 bp hits) to the Atlantic salmon chromosome sequences.

The assembled genome was also validated using CEGMA program (version 2.5) [24], by aligning 248 ultra-conserved eukaryotic proteins to resulting Atlantic salmon chromosomes. Since CEGMA proteins are highly conserved, alignment algorithms can identify their exon-intron structures on the assembled genome, thus allowing estimation of the completeness of the assembled genome in terms of gene coverage. CEGMA analysis (Supplementary Table 5) shows that 100% of CEGs proteins are present in our assembled genome and 81% of these were classified as complete. Collectively these unrelated analysis (EST, trout alignments and CEGMA) evidence that the assembled Atlantic salmon genome is highly complete in terms of protein-coding sequence.

Supplementary Table 5. CEGMA analysis of Atlantic salmon ICSASG_V2 at chromosome level (GenBank assembly accession number GCA_000233375.4). Total row represents the number of 248 ultra-conserved CEGs detected in the genome. A protein is classified as complete if the alignment of the predicted protein to the HMM profile represents at least 70% of the original KOG domain, otherwise is classified as partial. Groups 1-4 are different subset of CEGMA proteins being Group 1 the least conserved of all CEGs proteins and Group 4 the most conserved.

	**Complete**		**Partial**	
	Number Proteins	Percentage Completeness	Number Proteins	Percentage Completeness
Group 1	50	75.76	66	100
Group 2	45	80.36	56	100
Group 3	49	80.33	60	98.36
Group 4	56	86.15	65	100
**Total**	**200**	**80.65**	**247**	**99.6**

Increased read alignment depth and shorter scaffolds were characteristic of regions exceeding 95% similarity, representing 210 Mb (9.4% of the chromosome-positioned sequence), indicating assembly collapse (Figure 2). To evaluate quality of scaffolds within different regions in Atlantic salmon genome we selected the 70 longest homeologous blocks from Supplementary Table 6. Sequences within these blocks were split into 1 Mb bins and the average LASTZ % identity recorded for each bin (as shown in Figure 2). We enumerated 1013 Mb-bins from the first chromosome segment (lowest chromosome number) in all 70 blocks and analyzed the relationship between LASTZ identity, sequence variants and scaffold lengths. The assignment was done by overlap, meaning that short scaffolds in the interior of an Mb-bin were assigned to a single Mb-bin while longer scaffolds were assigned to multiple Mb-bins. Scaffold lengths are reduced markedly in Mb-bins with high LASTZ %ID, as shown in Extended Data Figure 6. In regions with average LASTZ %ID around 85 the median length of scaffolds is over 1 Mb, but the median length drops to just above 1Kb at the highest %ID, suggesting that the assembly collapses in the regions with the highest homeologous sequence identity.

2. Resolving homeologous regions in the salmon genome

To characterize the duplicated genome structure, and investigate mechanisms of rediploidization in different regions of the salmon genome, we aligned Atlantic salmon chromosome sequences using LASTZ[25] to disentangle conserved collinear blocks of homeology. LASTZ command line script; --targetcapsule=LZ_target_capsule query.fa —nochain --gfextend --nogapped —identity=75.0..100.0 —matchcount=100 —format=general —rdotplot=plotoutput.txt. In total 98 blocks, amounting 2.1 Gb (95%) of chromosome sequences, were identified (Supplementary Table 6). The conserved

synteny of Ss4R gene duplicates in these conserved homeolog blocks were very high (mean spearman cor. = 0.96).

The remaining sequence (120 Mb) could not be matched to a homeolog region with high colinearity suggesting that rearrangements, deletions, and/or fragmentation has preferentially eroded in some chromosome regions. The majority of these unmatched sequences are located at chromosome ends or at the sites where there is evidence for ancestral chromosome fusion (see red rectangles in Figure 2), suggesting that substantial amount of sequence has been lost in these regions.

Sequence similarity between homeologous sequences were determined in 1Mb interval by averaging local percentage of nucleotide sequence identity using High-scoring Segment Pair (HSP) from LASTZ alignments[25]. The alignments of sequence within homeolog blocks reveal that the salmon genome exists broadly in three states of regional similarity “normal” (≈87%), “elevated” (90-95%), and “high” (>95%) (Supplementary Table 7). Approximately 363.2 Mb (15%) of the salmon genome show sign of delayed rediploidization, characterized by elevated sequence similarity between homeologs, whereas close to 210 Mb (10%) may still retain residual tetrasomy by displaying a sequence similarity between homeologs higher than 95%.

Supplementary Table 7. Grouping of salmon genome into regions with different regional sequence similarity.

	**High (>95%)**	**Elevated (90-95%)**	**Normal (~87%)**	**Telomeric**	**Gaps**
**Genomic sequence**	209,995,658 (9.38%)	363,158,824 (16.22%)	1,354,347,153 (60.47%)	209,214,373 (9,34 %)	102,864,419 (4.59%)
**Genes**	4,938 (13.30%)	6,905 (18.60%)	20,941 (56.40%)	3,016 (8.12%)	1,330 (3.58%)

Contigs and scaffolds with elevated sequence similarity between homeologs are not randomly distributed in the salmon genome but coalesce into seven larger regions; 2p-5q, 2q-12qa, 3q-6p, 4p-8q, 7q-17qb, 11qa-26, 16qb-17qa, and to some extent 9qc-20qb and 5p-9qb. This phenomenon is exemplified by the comparison of ssa07 with its homeologous counterparts on chromosomes 17qb and 18qb in Extended Data Figure 7.

3. Salmon repeats

The Atlantic salmon repeat library contains a total of 2,005 repeat consensus sequences of which 1,093 (54.5%) are classified. Putative repeat sequences were obtained from *de novo* repeat-finding programs and existing databases prior to redundancy removal, host gene detection and classification.

3.1. *Preliminary repeat libraries*

Previously published transposable element (TE) repeat sequences were obtained from three sources: i) salmonid non-LTR retrotransposons reviewed by Matveev and Okada ([26]; nine sequences); ii) repeats identified in the rainbow trout genome ([21]; 634 sequences); and, iii) repeats in the RepBase database from Salmonidae species as of February 2015 ([27]; 366 sequences).

Three *de novo* repeat-finding programs were used to identify repetitive sequences in the genome: REPET v1.3.9[28], RepeatModeler v1.0.8[29] and LTRharvest, which is included within GenomeTools v1.5.1[30,31]. Two REPET libraries containing 581 and 919 sequences were produced using contigs longer than 10 Kb from two early draft Atlantic salmon genome assemblies. A single library of 927 sequences was produced by RepeatModeler using all of the contigs in Atlantic salmon genome assembly v3.6.

Two LTRharvest libraries were created and processed based on the "Repeat Library Construction – Advanced" instructions by Dr. Ning Jiang published on the MAKER wiki[32]. The libraries consisted of either recently active full-length long terminal repeat (LTR) retroelements or more ancient LTR retroelements. A putative LTR element was included in the recent library if its terminal repeats were at least 99% similar to each other and included in the older library if its terminal repeats were at least 85% similar. All LTR elements were required to possess either a poly-purine tract or primer binding site identified using the LTRdigest tool packaged within GenomeTools v1.5.1[33]. Primer binding site detection was assisted by a library of eukaryotic tRNA sequences obtained from the Genomic tRNA Database[34]. For each sequence, alignments of the 50 bases upstream and downstream of each LTR were performed in order to identify and exclude common false positives caused by tandem local repeats, local gene clusters or adjacent TEs. If the aligned upstream and downstream sequences had at least 25 identical bases and were at least 60% similar to each other the entire element was excluded[32]. Tools from BLAST+ v2.2.28[35] were used to identify high-quality reference elements and reduce redundancy. Elements containing nested insertions of other non-LTR TEs were detected and removed based on the presence of a good (E-value ≤ $1e^{-10}$) TBLASTN high-scoring segment pair (HSP) when compared to a TE protein library (REPET-formatted RepBase v19.06) that had all LTR retrotransposon-like proteins removed. Both BLASTN and RepeatMasker v4.0.5[23] were used to reduce redundancy within and between the two LTR element libraries using a cutoff of 80% identity over 90% of the element length. In order to identify less-similar and longer HSPs all BLASTN searches that were performed in this and other steps were made using a word size of 7 bp and without the low-complexity (dust) filter. Finally, the two libraries were combined and BLASTN was used to identify and count fragments of each LTR element in the genome. Only sequences producing 10 or more 500 bp HSPs were retained leading to a final LTRharvest library containing 207 sequences.

3.2. Removing redundant, low-repetition and chimeric sequences

Putative repeats from the two REPET libraries, the RepeatModeler library and the trout genome library were assigned a confidence level based on the length and number of BLASTN hits on unique contigs in the Altantic salmon genome. Any sequences that generated three or more HSPs at least 80% of their length were designated as *high confidence* (HC). Sequences not designated as HC were classified as *lower confidence* (LC) if they produced 10 or more hits of at least 100 bp; otherwise they were eliminated. Sequences from Matveev & Okada's TE library and RepBase were assumed to be well-curated and were automatically included in the HC library.

Most LC sequences failed to generate long (80%) HSPs due to a stretch of ambiguous bases or the inappropriate concatenation of separate repeats by the source *de novo* repeat-finding program. Such chimeric sequences were generally composed of two or more distinct repeats joined by non-repeated sequences. To reduce the possibility of chimeras the number of long HSPs (80+ bp) overlapping each LC sequence base was determined using BLASTN and the sequence was split wherever the HSP coverage dropped below 10 over 10 consecutive bases, with low-coverage sequence being removed.

Based on the guidelines established by ref.[36] redundancy was reduced within each of the HC and LC libraries by removing a sequence A if there existed a longer sequence B such that A was at least 80% covered by long (80+ bp) non-overlapping HSPs from B possessing at least 80% identity. Sequences in the LC library were also removed if they possessed a match to a sequence in the HC library using the same criteria.

The LTRharvest library was used to mask both the HC and LC libraries. RepeatMasker was run with an 80% minimum similarity threshold and any HC or LC sequence that was at least 90% masked was eliminated. The remaining HC and LC sequences were combined with the LTRharvest elements to produce a library containing 2,041 sequences.

3.3. Host gene detection and removal

In order to identify and remove non-TE host genes the merged library was compared to both the SwissProt UniProtKB database[37] and a TE protein database made of REPET-formatted RepBase v19.06 sequences as well as all RepBase Actinopterygii sequences as of March 2015 (many of which were absent from the REPET-formatted version). Host genes were generally identified as those repeats having a strong (E-value $\leq 1e^{-10}$) BLASTX hit to a non-TE UniProtKB protein while not possessing a hit to a RepBase TE protein with a better bit score. However, in cases where the two bit scores were very similar the sequences were manually reviewed. A total of 36 repeat sequences were classified as putative host genes and removed from the library.

3.4. Repeat classification

Classification of repeat sequences was based on the guidelines established by[36]. The PASTE Classifier tool included with REPET v2.0 was used in combination with BLASTN and BLASTX to identify structural motifs and to establish similarity to reference sequences for classification. Reference databases consisted of: i) a REPET-formatted set of PFAM HMM Gypsy profiles available on the REPET website (v26.0); ii) nucleotide and protein sequences from the REPET-formatted RepBase v19.06 library; iii) all proteins from RepBase TEs found in Actinopterygii species as of March 2015; iv) all nucleotide sequences from RepBase TEs found in Actinopterygii species as of May 2015; v) eukaryotic rRNA sequences from release 115 of the SILVA rRNA database[38]; and, vi) salmonid SINE and LINE retroelement sequences reviewed by Matveev and Okada.

Repeat library sequences were classified to the family level using BLASTN if, when compared to a nucleotide reference sequence, at least 80% of their sequence was covered by long (80+ bp) non-overlapping HSPs with greater than 80% similarity. If no family-level hit existed sequences were classified at the superfamily level based on their best BLASTX hit to a reference TE protein (E-value ≤ $1e^{-10}$). LTRharvest elements without a superfamily-level categorization were classified as being in the LTR order and sequences identified as miniature inverted-repeat elements (MITEs) by PASTE Classifier were assigned to the TIR order. All sequences were analyzed by dot plot to identify and classify those consisting solely of a tandem repeat (satellite) motif. Library sequences with conflicting classifications and those labeled as 'PotentialChimeric' by PASTE Classifier were manually examined and, if an obvious category could not be established, were labeled as 'Unknown'. Results from repeat classification are listed in Supplementary Table 8.

3.5. Tc1-Mariner activity history

Historical patterns of activity for elements of the most abundant TE superfamily in the Atlantic salmon genome, Tc1-Mariner, were reconstructed by comparing the amount of sequence divergence between TE instances within an individual family. A set of full-length representative sequences was determined for 40 Tc1-Mariner families, each of which was confirmed to be phylogenetically distinct from the others by alignment with MUSCLE v3.8.31[39] and subsequent construction of a NJ tree using MEGA5[40]. For each family representative sequence, BLASTN was used to obtain up to 100 randomized genomic instances that were each required to be at least 60% of the length of the representative. Instances were aligned using MUSCLE and the nucleotide pairwise percent similarity was calculated between each sequence pair. Historical patterns of Tc1-Mariner activity were visualized using a stacked density plot of the pairwise distances between instances of a family scaled by RepeatMasker-estimated genomic abundance. As with other Tc1-Mariner TEs all but the most recent families possessed star-like NJ tree topologies indicating a sudden period of rapid expansion followed by a period of inactivity and neutral mutation accumulation[41].

Supplementary Table 8. Repeat content of the Atlantic salmon genome. The genomic abundance of Class I and Class II TE taxa was parsed from RepeatMasker output and is not necessarily additive (individual repeat annotations can slightly overlap). RepeatMasker associated 50.03% of the genome with interspersed repeats and masked 59.89% of the genome as repeat-derived content.

Repeat Type	Order	Superfamily	Coverage (Mbp)	Coverage (%)
**Class I TEs**	**All**	**All**	**636.07**	**20.72**
	**LTR**	**All**	**180.11**	**5.87**
		Gypsy	84.25	2.74
		ERV	25.51	0.83
		Copia	7.77	0.25
		Bel-Pao	3.01	0.1
	**DIRS**	**DIRS**	**2.64**	**0.09**
	**PLE**	**Penelope**	**10.02**	**0.33**
	**LINE**	**All**	**422.24**	**13.76**
		Rex1	128.4	4.18
		Crack	114.04	3.72
		L2	109.38	3.56
		RTEX	26.5	0.86
		Tx1	20.52	0.67
		L1	14.21	0.46
		Nimb	5.19	0.17
		Jockey	1.96	0.06
		R2	0.89	0.03
		Togen	0.6	0.02
		CR1	0.32	0.01
		RTE	0.23	0.01
	**SINE**	**All**	**21.06**	**0.69**
		tRNA	19.26	0.63
		Deu	1.8	0.06
**Class II TEs**	**All**	**All**	**605.57**	**19.73**
	**TIR**	**All**	**597.96**	**19.48**
		Tc1-Mariner	395.79	12.89
		hAT	81.94	2.67
		piggyBac	7.47	0.24
		CMC-EnSpm	5.25	0.17
		Ginger	4.24	0.14
		PIF-Harbinger	3.14	0.1
		Sola	2.39	0.08
		IS3EU	1.8	0.06
		Kolobok	0.54	0.02
		Dada	0.17	0.01
	**Crypton**	**Crypton**	**6.08**	**0.2**
	**Maverick**	**Maverick**	**1.53**	**0.05**
**Unclassified**			**372.87**	**12.15**
**Tandem Repeats**			**223.39**	**7.28**
**Simple repeats**			**71.1**	**2.32**

4. Gene Annotation

4.1. Identification of gene structures

An automated pipeline for protein coding gene annotation was used to build gene models from spliced reference sequence alignments of short reads from Illumina platforms and longer EST/mRNA sequences (Supplementary Table 9). RNA-seq short reads were trimmed using Trimmomatic (v0.32, [42]) with the parameters (ILLUMINACLIP:TruSeq3-PE-2.fa:2:30:10:8: true

LEADING:3 SLIDINGWINDOW:20:20 MINLEN:40) and mapped to the reference genome sequence using STAR (v2.3.1z12, [43]) while long mRNA sequences downloaded from NCBI were mapped with GMAP (2014-07-28, [44]). Spliced alignments for each tissue were used to predict transcripts using cufflinks, with --multi-read-correct, and finally merged using cuffmerge.

Open reading frame (ORF) prediction were carried out using TransDecoder (http://transdecoder.github.io/,[45]) using the pfamA and pfamB databases for homology searches (--search_pfam) and a minimum length of 30 amino acids for ORFs without pfam support (-m 30). In addition to the pfam homology evidence we also made a BLASTP (evalue<1e-10) for all predicted proteins against *Danio rerio* (v9.75) and Gasterosteus aculeatus (BROADS1.75) annotations downloaded from Ensembl. Only gene models with support from at least one type of homology search (pfam or BLASTP) were kept.

We used Blast2GO[46] against the SwissProt database with default settings to predict functional annotation for predicted protein coding genes. The search retrieved 46,032 blast hits with 38,443 hits having a GO annotation. Transposable element related ORFs were identified in two ways. First, a curated list of annotation keywords related to repetitive elements (e.g. transposon, long terminalrepeat, etc) was then used to identify loci for which >=50% of the annotation descriptions matched the repeat element keyword list. Next, we additionally queried the Blast2GO annotation gene names for TE-related terms (i.e. retrotransposon, transposon, transposable, transposase, reverse transcriptase, gag, bpol).

In total we mapped 1.93 billion RNA-seq and EST/mRNA sequences to the genome (Supplementary Table 9). After merging transcript models using cuffmerge we were left with 128,426 transcribed loci which were used to predict open reading frames. Supplementary Table 10 shows the results from TransDecoder and homology support filtering of putative protein coding loci.

Supplementary Table 10. Summary of open reading frame detection with TransDecoder.

**Annotated**	**Number**
Total gene loci	88,182
Total transcripts	253,374
Loci w/ homology support	55,620
Transcripts w/ homology support	199,861
Complete gene models w/ homology support	44,730

In total, 55,620 putative protein coding genes were identified, of which 54,692 genes (98%) were anchored to a chromosome position. After removing the putative TE-related loci, the mean length of the longest transcript variant CDS were 1,145 bp, the mean number of alternative transcripts per locus were 4.2, with 69% of all loci having at least two transcript variants. Further filtering of gene loci, keeping only those genes having a SwissProt annotation (total 37,206 genes), increased the mean length of the longest transcript variant CDS to 1,232 bp while the mean number of alternative transcripts per locus were 4.8, with 81% of all loci having at least two transcript variants (Extended Data Figure 8a and b). Based on output from TransDecoder 80% (29,645) of these 37,206 highly reliable gene structure annotations were classified as complete, meaning they had a well defined 5'UTR, start and stop codons, as well as a 3' UTR. The remaining 7,561 genes classified as being partial gene structures were subdivided as follows: 5' partial genes = 14%, 3' partial = 4%, and internal=2%.

We then compared the annotated gene set excluding putative TE-related genes with well established annotations of stickleback and zebrafish from Ensembl. Compared to the stickleback annotation the salmon annotation contained more putative zebrafish orthologs (Extended Data Figure 8c), as well as comparable BLASTP statistics (Extended Data Figure 8d-f).

4.2. Classifying Ss4R gene duplicates

We identified expressed and silenced gene duplicate loci (i.e. homeologs) originating from the Salmonid whole genome duplication using a multi-step approach (see details in Extended Data Figure 8g). Expressed homeologs were identified using BLASTP and TBLASTN searches. Silenced loci were identified using a combination of GenomeThreader[31], TBLASTN, and BLASTN.

Supplementary Table 11 shows the number and proportion of genes having an expressed or silenced homeolog gene copy using different homeolog aligning strategies. Based on these data we estimate genome wide percent conserved and expressed Ss4R gene duplicates to at least 50-60%. This is an underestimate as the genomic regions with extremely high sequence similarity between homeologs will be collapsed in the assembly.

Supplementary Table 11. Classification of homeolog gene duplicate pairs.

Gene set	Gene numbers	Genes w/ expressed homeolog			Genes w/ silenced homeolog				Singletons*
		Total expressed duplicates	BLASTP duplicates	TBLASTN duplicates	Total silenced duplicates	GTH silenced	TBLASTN silenced	BLASTN silenced	
All loci	**55620**	**25548** (45.93%)	**21090** (37.92%)	**4458** (8.02%)	**5839** (10.5%)	**881** (1.58%)	**1847** (3.32%)	**3111** (5.59%)	**24233** (43.57%)
Excl. TEs	**46598**	**25548** (54.83%)	**21090** (45.26%)	**4458** (9.57%)	**5839** (12.53%)	**881** (1.89%)	**1847** (3.96%)	**3111** (6.68%)	**15211** (32.64%)
Excl. TEs Incl. SwissProt	**37206**	**22803** (61.29%)	**18624** (50.06%)	**4179** (11.23%)	**5125** (13.77%)	**808** (2.17%)	**1567** (4.21%)	**2750** (7.39%)	**9278** (24.94%)

**No evidence of homeolog copy (neither expressed nor silenced) in the expected homeolog chromosome region.*

5. Evolution and retention of Ss4R homeologs

5.1. Estimation of ortholog/homeolog gene trees

We constructed ortholog sequence sets which included orthologs/homeologs from Atlantic salmon (CIGENE annotation v2.0), rainbow trout[21], and the diploid outgroup genomes of *Esox lucius* (CIGENE annotation v1.0), *Danio rerio* (v9.75), and *Gasterosteus aculeatus* (BROADS1.75) as outgroups.

Initial grouping of gene sequences into ortholog sequence sets were done by a best reciprocal blast (BRB) strategy using the *E. lucius* annotation as a reference. For the partially tetraploid salmonid species top-two BRB-hits were assigned to putative otholog groups. All Gene tree analyses were carried out using an early version of the gene annotation (v2.0). This version differed by not including the samples from developmental stage and using scaffolds as reference sequences rather than the chromosome pseudomolecules.

Multiple sequence alignments were constructed for each ortholog sequence group using MAFFT[47] and quality trimmed with Guidance in an iterative framework, as described in ref.[48]. In brief, Guidance is using MAFFT to generate sequence alignments using codon-based models. Each alignment is then bootstrapped 100 times and column (i.e. codon) scores and sequence (i.e. gene sequence) scores are calculated based on the alignment reproducibility across bootstrap replicates. Any sequence with a Guidance sequence score <0.8 are removed from the input sequence files and a new alignment with reduced sequence numbers is calculated. This is iterated until all sequences in ortholog groups pass the sequence score threshold. Only columns (codons) passing default Guidance score cutoff was used in the final alignments.

Maximum likelihood (ML) gene trees were calculated in the R-package Phangorn[49] using the GTR+G+I model and 100 bootstrap replicates. Only trees being monophyletic for

Esox+salmonids and *Salmo+Oncorhynchus* as well as having bootstrap support of >=50% for all nodes were considered in the final analyses. Due to the deep (i.e. old) divergence between *D. reiro* + *G. aculeatus* and *Esox* + Salmonids, *D. reiro* and *G. aculeatus* were removed from all gene trees prior to analyses of sequence evolution.

Initial BRB-analyses identified 13,672 ortholog+homeolog sequence sets. These were further reduced to 5,935 after filtering for sequence alignment robustness, minimum bootstrap support of 50%, minimum two salmonid gene sequences per gene tree, and monophyletic salmonid clade with a *E. lucius* gene sequence as an outgroup (Supplementary Table 12).

Supplementary Table 12. Ortholog group summary results. BRB = raw results from best reciprocal blast analyses. All topologies = number of gene trees including gene sequences prior to filtering. Final topologies = final gene trees data used for analyses.

Species	Ortho-groups		
	BRB un-filtered	All topologies	Final topologies
E. lucius	13672	9480	5935
D. reiro	9761	8183	NA
G. aculeatus	8623	7673	NA
S. salar 1	10327	9052	5776
S. salar 2	7034	6281	3647
O. mykiss 1	9732	8438	5434
O. mykiss 2	6723	5688	3403
All	13672	9514	5935

All 5,935 gene trees could be classified into 13 different topologies (Extended Data Figure 9c) where the largest class (2,114 trees) was gene trees having two homeologs in both salmon and rainbow trout and a common duplication node. This means that most duplicated loci in our data set underwent diploidization prior to salmon-rainbow trout divergence.

We assessed the reliability of ortholog/homeolog gene tree dataset by counting homeolog loci from known duplicated regions originating from the salmonid whole genome duplication (Extended Data Figure 9d). Ninety six percent of the duplicates in gene trees were from known homeolog regions, indicating a reliable gene tree-based homeolog definition. Only about 0.9% of gene trees contained duplicates on the same chromosome (i.e. likely segmental duplications not related to the salmonid whole genome duplication).

5.2. *Comparative gene tree content analyses*

Gene trees were ~25% more likely to contain a duplicated homeolog from Atlantic salmon compared to rainbow trout (Fischer test: Odds ratio=1.257, 95% confidence interval=1.16-1.36, Counts: Atlantic salmon = 3,580 duplicated, 2263 singletons; Rainbow trout = 3,162 duplicated, 2,513 singletons). This could be due to true biological differences in duplicate loss rates (silencing) of homeologs or it could be related to technical artifacts, for instance frequency of genome assembly collapse of highly identical regions (i.e. genomic regions still behaving as a tetraploid) or gene annotation differences.

5.3. *Signatures of Salmo-specific rediplodization or residual tetrasomy*

Three gene tree topology classes reflected signatures of maintenance of tetrasomic inheritance after the divergence between rainbow trout and salmon. The level of genomic similarity (at 1 Mb scale) is much higher at loci reflecting tetrasomic inheritance in Atlantic salmon after the *Salmo-Oncorhynchus* divergence compared to the homeolog loci having topologies reflecting rediploidization in a salmon and rainbow trout ancestor (Extended Data Figure 9c).

5.4 *Analyses of sequence evolution*

Gene trees with homeolog copies in both Atlantic salmon and rainbow trout were analysed for sequence evolution rate asymmetry following the salmonid whole genome duplication. By including rainbow trout in the gene tree analyses we could partition the analyses into (i) ancestral branches (shared evolution between *Salmo* and *Oncorhynchus*) and (ii) *Salmo/Oncorhynchus*-lineage specific branches. Three rate estimates were calculated using maximum likelihood methods; GTR-model DNA sequence rates, synonymous substitutions per synonymous sites (dS), and non-synonymous substitutions per non-synonymous sites (dN). The GTR rate estimates were directly inferred from gene tree branch lengths using a combination of custom R-scripts and functions from the R-package ape. dS and dN rates were inferred indirectly from pairwise synonymous and non-synonymous distance matrices calculated by codeml in the PAML software package[50]. Non-negative least squares regression were then used to estimate dS- and dN/dS-rates for gene trees branches using the nnls.tree() function, combining the distance matrixes and ML-topologies, in the R-package Phangorn[49].

We assessed the evolutionary consequence of the salmonid genome duplication on the sequence evolution rates of duplicated genes. Functional redundancy of duplicated genes can result in relaxation of purifying selection pressure of one or both of the gene duplicates and ulitmately adaptive amino acid sequence level changes[51]. Such sequence level process are expected to leave signatures of increased rates of non-synonymous substitutions and hence an increase of the ratio

between non-synonymous and synonymous substitutions per site (dN/dS=ω). Under relaxation of purifying selection in one homeolog (but no positive selection) difference in GTR-rates and ω will increase and approach ω=1, while under positive selection codons under selection is expected to have a ω>1.

Differences in rate asymmetry, quantified as difference dN/dS rates along *Salmo+Oncorhynchus* ancestral branch and lineage specific tip branches, showed that asymmetry homeolog sequence were significantly higher after the divergence of *Salmo* and *Oncorhynchus* lineages (Supplementary Table 13, Extended Data Figure 9d). Similar pattern was also found when comparing GTR rates. As expected by the neutral theory, there was no homeolog rate asymmetry in dS rates.

Supplementary Table 13. Resampling test statistics for differences in Ss4R rate asymmetry between ancestral and Salmo-lineage branches. The 0.01% and 99.9% quantiles for resampling differences in the median branch length ratios: (fastest-ancestral/slowest-ancestral)-(fastest-lineage/slowest-lineage).

Rate-type	Quantile-low	Quantile-high
	0.01%	99.9%
GTR	-0.19	-0.06*
dS	-0.2	0.03
dN/dS	-0.03	-0.02*

* significant at p<0.001

These rate asymmetry patterns suggest that different evolutionary forces have acted on homeologs prior to- and after the divergence between *Salmo* and *Oncorhynchus*. One interpretation is that positive selection has been a stronger force subsequent to the radiation of *Salmo* and *Oncorhynchus*, suggesting that an increase in homeolog rate asymmetry is linked to the independent adaptation to different environments during species radiation of *Salmo* and *Oncorhynchus* lineages. However, from analyses of homeolog divergence times (i.e. signals of diploidization after whole genome duplication) it is also evident that non-homeolog crossing over occurred for at least 20 million years after the whole genome duplication. This process would certainly have counteracted and eroded asymmetric accumulation of substitutions during early evolution of the salmonid lineage.

5.5. Test for positive selection

Branch-site specific test for positive selection was carried out using codeml in PAML. For each tree, the likelihood of two competing ML-models was estimated: first model allowing for positive

selection on a pre-defined branch/set of branches, and a second model restricting sequence evolution to neutral (ω=1) or purifying (ω<1) selection. The two models are finally evaluated using a likelihood-ratio test (LRT). Likelihood estimations for models allowing for positive selection were run with four separate omega starting values (0.5, 1, 1.5, and 2). The result giving the lowest likelihood was used in the LRT. False discovery rate adjustments of p-values were done with the p.adjust function in R.

Tests for positive selection on branches with highest GTR and dN/dS rates revealed that 123 (5.9%) and 112 (5.4%) loci had a significant test for positive selection at lineage specific branches, respectively. The loci with significant tests results were 96.4% overlapping (108 loci overlapping). Similar statistics for ancestral branches were 7 (0.03 %) and 3 (0.01 %) significant tests for positive selection using GTR- and dN/dS-asymmetry to define the fastest evolving branch (3 overlapping test result).

We also constructed hypothesis test for positive selection for which both salmon homeologs evolved under positive selection. A similar proportion of loci (108 out of 2,084) as the 'fastest-branch tests' had signatures of positive selection; however, the overlap between loci under positive selection was only 77%.

6. BEAST dating of homeolog rediploidization times

6.1 Gene tree calibration

We used BEAUti (v1.7.4,[52]) to generate a single dummy input file for BEAST analyses with a specified HKY+G substitution model, uncorrelated lognormal clock, and yule tree prior. Using custom R-script alignments were then added to the xml dummy input file to generate a single xml-file for each orthogroup with two salmon and two rainbow trout homeologs. The same alignments as used in the ML-gene tree estimation was used in the BEAST analyses, only with small modifications to number of taxa included. Firstly, only orthogroups having both *G. aculeatus* and *E. lucius* as salmonid outgroups were used. Secondly, all *D. reiro* sequences were omitted from alignments. A single secondary calibration of 127 million years (MY) (confidence interval 12.5 MY) on the most recent common ancestor of Salmoniformes + Esoxiformes were used[53,54]. All analyses were run for 10 million generations with sampling every 1000 generations. No priors on tree topology were specified. Tracer[55] Tracer v1.6, Available from http://beast.bio.ed.ac.uk/Tracer) was used to inspect effective sample sizes (ESS) of tree parameters.

BEAST '.tree' files were used to generate 50% consensus topologies of 100 randomly sampled tree topologies from the last 1000 MCMC-samples.

6.2 Analyses of divergence times

Log files from MCMC-sampling was used to estimate median node age estimates for the most recent common ancestor (tmrca) nodes of taxa using custom R-scripts. The salmonid homeolog divergence age was estimated by sampling tmrca age for the two salmon and two rainbow trout homeologs, respectively. Age of speciation was estimated as the median of the both tmrca nodes. Hence, we have two estimates of speciation age and two estimates of the homeolog divergence for each gene tree.

1,671 gene trees passed the filtering criteria for tree topology reflecting rediploidization prior to *Salmo-Oncorhynchus* divergence (Extended Data Figure 2a). Manually inspection of 10 random trees showed that 10 million MCMC-generations produced sufficiently large ESS of tree parameters, most often in the range of 1000-3000.

A clear correlation between homeolog divergence age and genomic sequence similarity validates the use of % similarity between homeolog genome sequence as a proxy for time (Extended Data Figure 2b). Extended Data Figure 2c show the distribution of gene tree divergence times between *Salmo-Oncorhynchus* and Ss4R homeologs. The modes of the distributions are indicated as vertical lines and represents point estimates for *Salmo-Oncorhynchus* divergence time (20.9 MYA) and homeolog rediploidization age (76.9 MYA). The homeolog divergence mode should be interpreted as the age at which the majority of the duplicated genes reverted back to function as diploid loci. The whole genome duplication event must therefore has occurred between the split between Esoxiformes and Salmoniformes (127 MYA, 95% CI = 12.5 MYA) and ~80 MYA.

One confounding factor of the gene tree calibration is the history of non-homologous recombination within homeologs in gene trees. Homeolog loci that have evolved for a long period without returning to a true diploid state will have increased probability of having undergone non-homologous recombination event between different homeoloci. This process will affect the estimated number of substitutions and the parameter estimates of the evolutionary model. Extended Data Figure 2d shows some correlation between *Salmo-Oncorhynchus* node age and homeolog divergence age. This correlation supports the idea that non-homologous recombination can have confounded the node age estimates and inflated the variation in age estimates. We therefore normalized the homeolog divergence age estimates by fixing the *Salmo-Oncorhynchus* divergence to 21 MYA (see Figure 3 in main article).

7. Analyses of transcriptome

7.1. Tissue specific Expression in salmon

Gene expression was quantified with RNA-Seq in 15 tissue samples from wild type (WT) salmon and 10 tissue samples from the double haploid Sally. 39,799 of 55,467 genes had an FPKM

expression value above 1.0 in at least one sample (i.e. henceforth referred to as "expressed"). FPKM expression values were transformed to log2 (FPKM+1) values for downstream analysis. Samples and genes were clustered using Pearson correlation and Ward's method in the R function hclust[56]. Dendrograms and heatmaps were generated using the R function heatmap.2 in the gplots library. Genes were scaled.

Hierarchical clustering revealed that most of the common tissues between WT and Sally were clustered together (Extended Data Figure 4a). We therefore focused on the WT samples with higher read-coverage in the preceding analysis, where 38,015 genes were expressed. 11 gene clusters (named A - K) were identified visually using the dendrogram (Extended Data Figure 4b).

7.2. Evolution of homeolog gene regulation

3,991 out of 8,954 (45%) expressed homeolog pairs (10,774 pairs in total) showed signs of diverged expression by being located in different expression clusters. To identify specific patterns of divergence, we identified cluster-pairs with significantly many shared homeolog-pairs. Specifically, we computed p-values for each cluster-pairs as the fraction of 10,000 randomized clusterings that resulted in more shared homeolog-pairs than the original clustering (Supplementary Table 14).

Supplementary Table 14. Homeolog-pair in different co-expression clusters. 3,991 of 8,954 homeolog-pairs in different clusters. The numbers in the table are P-values associated with the numbers of homeolog-pairs shared between clusters (based on 10,000 permutations).

	B	C	D	E	F	G	H	I	J	K
A	0.0000	0.8307	0.9388	0.9997	0.8619	0.9999	0.1370	0.7523	0.5235	0.9882
B		0.0064	0.9842	0.7357	0.1399	1.0000	0.7598	0.0011	1.0000	0.8742
C			0.0189	0.0000	0.0158	0.4552	0.7971	1.0000	1.0000	0.9988
D				0.0000	0.0000	1.0000	1.0000	0.9597	0.9823	0.9490
E					0.0136	0.5154	0.9999	0.9911	1.0000	1.0000
F						0.9998	0.0340	0.0846	1.0000	1.0000
G							1.0000	1.0000	0.0000	1.0000
H								0.0000	0.8160	0.9861
I									0.9852	0.9981
J										0.0000

To further understand the divergence of homeolog expression, we utilized gene expression data from pike. Using the same definition as before, 26,844 of 29,049 pike genes were expressed based on 13 tissue samples (all overlapping with the 15 tissues from WT salmon; Skin and Ovary were not available). We analyzed 8,102 expressed triplets (9,112 triplets in total) containing a pike gene (denoted P) and the orthologous salmon homeolog-pair (denoted S1 and S2, where S2 had the highest expression similarity to P). In general, the salmon homeologs were more co-expressed with

each other than with the pike ortholog (Extended Data Figure 4c, p < 2.2e-16 and p = 7e-5, respectively, Wilcoxon signed rank test using the R function wilcox.test) indicating that there has been evolutionary pressure to maintain the expression of both homeologs.

In 5,666 of the 8,102 (70%) triplets, at least one homeolog (S2) had conserved expression in pike (P), defined as a significant Pearson correlation (>0.6, P = 0.03) across the 13 common tissues. Interestingly, 2,272 of these 5,666 (40%) conserved triplets contained salmon homeologs with diverged expression (i.e. S2 and P exhibited conserved expression while S1 and S2 belonged to different gene clusters using the same 11 clusters as before, neofunctionalization). Again we found significant patterns of divergence between the clusters by employing randomization (Supplementary Table 15). These patterns were visualized in a heatmap of the 2,272 triplets (main Figure 4d). In the heatmap, the triplets were first sorted by the gene cluster of S2 and then by the clustering order of S1. Each gene was scaled separately.

Supplementary Table 15. Homeolog-pair (with pike orthologs) in different co-expression clusters. 2,272 of 5,666 homeolog-pairs were in different clusters. The numbers in the table are P-values associated with the numbers of homeolog-pairs shared between clusters (based on 10,000 permutations).

	A	B	C	D	E	F	G	H	I	J	K
A		0.0000	0.8566	0.9599	0.9624	0.9707	0.9987	0.0132	0.7939	0.6848	1.0000
B			0.0049	0.8207	0.9560	0.2196	1.0000	0.7796	0.0014	1.0000	0.7657
C				0.1962	0.0000	0.0098	0.3869	0.8655	0.9988	1.0000	0.9992
D					0.0004	0.0019	0.9769	1.0000	0.8898	0.9111	0.4994
E						0.1016	0.4342	0.9999	0.9622	1.0000	0.9997
F							0.9087	0.0547	0.1544	1.0000	0.9999
G								1.0000	1.0000	0.0000	1.0000
H									0.0000	0.8704	0.9942
I										0.9995	0.9945
J											0.0000

One pattern that stands out in the triplet heatmap is that some homeologs appear to diverge in expression specificity. We computed specificity as one minus the sum, over all samples, of the gene’s expression in that sample divided by the maximum expression in any sample. A score close

to one indicate specific expression (~ the gene is expressed in one sample) while a score close to zero indicate broad expression (~ equally expressed in all samples). For homeolog-pairs with the conserved gene (S2) in the broadly expressed cluster (cluster G), the diverged gene (S1) tended to be more specialized by exhibiting a much more specific expression (in brain, ovary and testis, P = 1E-8, Wilcoxon rank sum test using the R function wilcox.test). For pairs with the conserved gene specifically expressed in the brain (cluster H), the diverged gene tended to be less specific (P = 6E-4). Finally, for pairs with the conserved gene specifically expressed in the eye (cluster I), the diverged gene, again, tended to be less specific (P = 7E-5) (Extended Data Figure 4d).

1,084 of the 8,102 (13%) triplets were candidates for subfunctionalization: both salmon genes had diverged from pike (Pearson correlation < 0.55, P > 0.05) and each other (different gene clusters). However, only 23 showed clear signs of subfunctionalization, where the sum of the salmon expression correlated with that of the pike ortholog (Pearson correlation > 0.6).

We also analyzed expression data on the level of exons in order to reveal regulatory divergence hidden at the gene level. Here we used Pearson correlation < 0.55 to define divergence between salmon homeologs rather than gene clusters. In 1,985 of the 5,932 (33%) homeolog-pairs with conserved expression at the gene level, and at least two homeologous exon-pairs, at least one exon-par had diverged in expression (33% exon-divergence). About one-third of this divergence was associated with exons with low or no expression; 22% exon-divergence was observed after removing lowly expressed exons (no FPKM values above 1.0). Noise due to high expression variability of short exons did not have a huge effect; 30% exon-divergence was observed after removing exon-pairs with a BLAST alignment shorter than 100 bp. 20% exon-divergence was observed after removing both lowly expressed exons and short exons. Again using pike as an outgroup (427 triplets), we classified exon-divergence into neofunctionalization (64%) and subfunctionalization (1%). These numbers were the same after filtering for low expression or exon length, and were also remarkably similar to what was observed at the gene level (63% and 1%, respectively)

By combining the gene-level divergence (45%) and exon divergence (30%), we estimated that approximately 63% of the homeolog-pairs in salmon showed signs of diverged expression.

To make sure that divergent expression between homeologs were not associated with signatures of pseudogenization we looked at the correlation between per cent divergence in CDS length and tissue expression correlation between the Ss4R duplicates. No large difference in CDS-length difference was apparent between Ss4R homeologs showing divergent or similar homeolog expression divergence (red points versus black points in Extended Data Figure 4e).

We also classified regulatory divergence using an ‘on’ or ‘off’ strategy (see Extended Data Figure 4f). We defined on as: Gene G1 is "on" compared to G2 in tissue T if the expression of G1

is above 1.0 (2.0 in pike) and (1) the expression of G2 is below 1.0 and (2) the expression in G1 is twice that of G2.

Given a triplet: S1, S2 (salmon) and P (pike):

- We classify a Ss4R homeolog pair as regulatory subfunctionalization when S1 was on compared to S2 in at least one tissue T1, and S2 was on compared to S1 in at least one other tissue T2 (this is only true in 676 homeolog-pars). And, finally, P is on in both tissues T1 and T2.
- We classify a Ss4R homeolog pair as regulatory neofunctionalization if S1/2 is on in at least one tissue compared to S2/1. And S1/2 is not on in any tissue compared to P, and P is not on in any tissue compared to S1/2, i.e. S1 or S2 is conserved in pike (5016 homeolog-pairs have one gene conserved in pike).

We identified 167 cases of regulatory subfunctionalization and 3028 cases of regulatory neofunctionalization. 1129 Ss4R pairs overlapped with correlation-based neofunctionalization (Hypergeometric test: 1129/2272, 3028/8102: $p = 9E\text{-}46$).

7.3. GO analyses of Ss4R with conserved and diverged gene regulation

We conducted a gene ontology (GO) overrepresentation analyses (using the R/Bioconductor package GOstats[57]) of genes that either had or did not have a retained expressed Ss4R duplicate.

We also tested the GO terms associated with retained Ss4R duplicates with similar or diverged expression regulation. In similarly expressed Ss4R duplicates, we found GO terms associated with genes having many interaction partners high up on the list of significantly overrepresented GO terms (Supplementary Table 16). This indicates that there is selection for maintaining stoichiometric balance through conservation of ancestral function in both Ss4R duplicates if the proteins have many interaction partners.

Supplementary Table 16. Over-represented GO terms for conserved gene regulation between Ss4R duplicates.

P-value	Odds raio	GOTerm
1,51307E-19	10,20	structural constituent of ribosome
2,3156E-12	1,45	nucleic acid binding transcription factor activity
2,56853E-11	1,33	DNA binding
1,47111E-10	0,80	small molecule metabolic process
2,40506E-10	2,59	ribosome
1,55695E-09	1,22	protein complex
4,27433E-09	1,20	nucleus
5,98777E-09	0,75	lipid metabolic process
1,49979E-08	2,25	ribonucleoprotein complex assembly
2,26707E-08	1,45	RNA binding
2,37008E-08	1,45	structural molecule activity
2,44819E-08	1,28	macromolecular complex assembly
3,67588E-08	1,24	cell morphogenesis
5,31049E-08	2,53	ribosome biogenesis
7,97986E-08	0,65	oxidoreductase activity
2,85124E-07	1,36	transcription factor binding
8,91435E-07	1,22	nucleolus
1,85449E-06	1,31	chromosome organization
3,34546E-06	1,21	cell-cell signaling
4,11067E-06	1,43	translation
9,98214E-06	0,77	carbohydrate metabolic process
3,62487E-05	3,71	rRNA binding
3,7484E-05	1,14	cellular nitrogen compound metabolic process
4,45681E-05	1,16	cellular component assembly
4,60667E-05	1,21	neurological system process
5,05356E-05	1,31	symbiosis, encompassing mutualism through parasitism
5,1961E-05	1,40	nuclear chromosome
6,2492E-05	1,40	mRNA processing
8,52482E-05	1,20	protein complex assembly
9,21422E-05	1,26	chromosome
9,23517E-05	0,69	sulfur compound metabolic process
0,0001	0,84	endoplasmic reticulum
0,0002	1,22	cytoskeletal protein binding
0,0002	1,25	protein binding transcription factor activity
0,0003	1,14	embryo development
0,0004	0,88	cytoplasm
0,0004	1,18	nucleoplasm
0,0006	1,14	cytoskeleton
0,0010	1,11	cell differentiation
0,0012	1,11	anatomical structure development
0,0012	1,14	cell cycle
0,0017	1,18	signal transducer activity
0,0029	0,87	mitochondrion
0,0030	0,73	cofactor metabolic process
0,0058	0,71	transferase activity, transferring glycosyl groups
0,0062	0,71	autophagy
0,0062	0,85	circulatory system process
0,0072	0,85	endosome
0,0074	0,86	kinase activity
0,0089	1,12	cytoskeleton organization
0,0109	0,62	hydrolase activity, acting on carbon-nitrogen (but not peptide) bonds
0,0133	0,69	lyase activity
0,0143	1,16	DNA metabolic process
0,0155	2,06	cell wall organization or biogenesis
0,0163	0,81	cellular amino acid metabolic process
0,0185	1,09	immune system process
0,0203	1,10	growth
0,0219	1,15	protein targeting
0,0220	1,45	small conjugating protein binding
0,0228	0,91	cell motility
0,0232	0,69	isomerase activity
0,0242	0,65	hydrolase activity, acting on glycosyl bonds
0,0244	0,93	plasma membrane
0,0244	0,74	ligase activity
0,0350	0,29	nitrogen cycle metabolic process
0,0387	0,85	extracellular matrix organization
0,0414	1,09	homeostatic process
0,0420	1,29	mRNA binding
0,0468	0,83	plasma membrane organization

8. Ts3R and Ss4R duplicate retention

8.1. Gene tree based duplicate retention analyses

To study the duplicate retention patterns of the autotetraploidization of the *Salmo salar* genome, an analysis of duplicate gene retention was performed. The analysis was performed by retrieving existing gene families from Ensembl Compara 79[58] for all teleost species. These gene families consisted of sequences from *Lepisosteus oculatus*, *Danio rerio*, *Astyanax mexicanus*, *Gadus morhua*, *Oreochromis niloticus*, *Gasterosteus aculeatus*, *Takifugu rubripes*, *Tetraodon nigroviridis*, *Oryzias latipes*, *Poecilia formosa*, *and Xiphophorus maculatus*. Genomes for *Salmo salar*, *Esox lucius*, and *Oncorhynchus mykiss* were then BLASTed against the Ensembl gene trees and themselves to determine homologous relationships using an e-value threshold of 1e-10. The homologous sequences were then aligned using MAFFT[59] (command line option –auto) to generate multiple sequence alignments. Quality control for matches involving salmon, trout, and pike included a match percentage of at least 50% identity for matched regions for Extended Data Figure 3 and also the fraction of pairwise aligned gaps of at most 50% for an added control analysis discussed here. A phylogenetic tree was built for each of the gene families using PhyML 3.4[60] using the JTT+G substitution model. The JTT+G substitution model was selected based on model selection results of OrthoMCL trees constructed using salmon, pike, and trout, which revealed the vast majority of gene families to be best fit under the JTT+G substitution model. The phylogenetic trees were then reconciled against the teleost species tree found in NCBI with *Lepisosteus oculatus* as the outgroup species (an inexhaustive sampling of trees that included gar were used). The reconciliation was performed using Softparsmap[61] which assigns duplications based on minimizing the number of inferred duplications within a tree using a parsimony approach. This allowed for duplication and speciation events to be assigned to each node in the tree, and to determine the correct root of the tree based upon the gar sequence. Trees beginning with a duplication event were subsequently split such that the oldest node for each tree was a speciation event. Ultimately, this resulted in generating 12,388 gene families, covering 26,325 salmon genes.

The constructed gene trees were then assessed for duplicate retention for the 3R WGD, 4R WGD, small scale salmon specific duplications (SSD) following the 4R event, and duplications occurring between the 3R and 4R WGDs. To determine how WGDs are retained and impact the retention of later duplications, we examined the retention of duplicates following the 3R, 4R, and salmon lineage specific duplications (Extended Data Figure 3). Duplicate retention was counted by examining the conditional percentages of genes that were retained from the 4R WGD following the 3R WGD and from the 4R WGD to small scale duplications on the salmon lineage. The duplication lineage for each gene was counted, ensuring that each lineage accounted for the retention or loss of a duplicate, with the expectation that each 3R duplication should give rise to two 4R WGDs, and

every 4R WGD should lead to two small scale duplications. Furthermore, duplications predating the 4R WGD should lead to two 4R WGD. Phylogenetic information from both the rainbow trout and salmon genes was used to assess differences between 4R and SSD duplications within the tree. Additionally to differentiate between ambiguous nodes, chromosomal locations were used to infer SSD duplicates when found on the same chromosome following a putative SSD. Although it was possible to distinguish the duplication type for many of the duplication events, some ambiguities did arise. For ambiguous duplication events that could have been either a 4R event or an SSD, if the lineage did not already have a 4R event present, the duplication was counted as a 4R event. If a 4R event was already present on the lineage, it was determined to be a post-4R SSD. Furthermore some duplication events, counted as being pre-4R duplications could potentially have been miscounted due to phylogenetic error within the tree from the misplacing of pike genes within the tree. These genes should putatively be assessed as 4R duplicates, but due to phylogenetic error were assessed as pre-4R duplications. Two variants of the code, one that rigidly used the phylogeny and another that was robust to local phylogenetic error were implemented. The data in Extended Figure 3 is derived from the error-robust variant. Furthermore, a minimal number of trees which demonstrated large deviations from the accepted teleost species tree were present in the dataset and were counted in the duplication analysis. These trees could potentially bias the conditional retention probabilities due to large numbers of duplications from potential phylogenetic errors within the trees. The duplication analysis was performed using custom perl scripts, which are available online. Conditional probabilities were then calculated to determine the likelihood of retention of a gene duplicate following each of the WGDs.

To further assess the retention of gene duplicates and how potential protein-protein interactions are impacted during the duplication retention process, protein-protein interactions for the salmon genome were built using the STRING database[62]. A BLAST of the salmon genome against *Danio rerio* was performed to determine homologs within the STRING database. STRING interactions were then determined for all genes within salmon which had homologs in *Danio rerio*, resulting in 46,966 putative homologs. STRING interactions were then parsed such that only interactions labeled "binding" were kept which are putative physical protein-protein interactions based on various forms of evidence. This resulted in ~7.7 million interactions for which a salmon homolog could be found for zebrafish. We then assessed duplication retention on protein-protein interactions and dosage compensation within a network (Extended Data Figure 3). STRING binding partners were examined to see if they were retained or lost following the same 3R WGD, 4R WGD, and SSD as the query partner. Retention counts for each duplication type were generated using the phylogenetic trees described above, where only genes in the subset of sampled trees were counted. The STRING interaction retention analysis was performed using custom perl scripts.

Upon completion of the duplication and retention of interacting partners analyses, statistical tests of significance were performed to determine if there was evidence to suggest that the duplication counts were significantly different from each other. The duplication process was represented by a binomial distribution where each duplication could have either been retained or not. We assessed the significance of each pair of retained duplicates at the pre-4R – SSD level, the 4R WGD level, and the post-4R – SSD level. For the post-4R – SSD duplications we examined the significance for the duplications retained after the 3R and 4R WGDs. Additionally we assessed the fraction of interacting partners that were retained after the 3R and 4R WGDs from the STRING retention analysis described above. In total seven tests were performed. A two-proportion pooled z-test was performed to calculate two sided p values at the Bonferroni corrected α-level of $\alpha < 0.001 / 7$. To further explore if the results seen were in fact significant with a marginal level of effect or being overly influenced by large sample sizes, an Odds Ratio and Relative Risk analysis was performed for each group and two sided p values where calculated. All tests showed extremely low p values indicating that the groups were significantly different from one another[63].

A table of results from different analyses is presented here.

Supplementary Table 17. A result of duplicate gene retention analyses with several different sets of assumptions is presented to show their effect.

doi:10.1038/nature17164

Salmon Duplication Analysis	is 3R Present?	Conditional Probabilities	is 4R Present?	Conditional Probabilities	is SSR Present?	Conditional Probabilities	is Pre4R Present?	Conditional Probabilities
Phylogeny relaxation with only threshold of percent identy 50%	Yes	20.02%	Yes	53.43%	Yes	10.43%	Yes	12.66%
			No	46.57%	No	89.57%	No	87.34%
					Yes	4.96%		
					No	95.04%		
	No	79.98%	Yes	54.83%	Yes	6.83%	Yes	4.22%
			No	45.17%	No	93.17%	No	95.78%
					Yes	4.03%		
					No	95.97%		
	Fraction of Interacting Partners Retained After 3R		Fraction of Interatcing Partners Retained After 4R		Fraction of Interacting Partners Not Retained Ater 3R		Fraction of Interacting Partners not retained at 4R	
	38.26%		74.98%		35.68%		73.90%	
	Fraction of proteins with no interacting partners retained at 3R		Fraction of proteins with no interacting partners retained at 4R					
	34.93%		65.98%					

Salmon Duplication Analysis	is 3R Present?	Conditional Probabilities	is 4R Present?	Conditional Probabilities	is SSR Present?	Conditional Probabilities	is Pre4R Present?	Conditional Probabilities
Phylogeny relaxation with no threshold	Yes	20.16%	Yes	53.00%	Yes	10.59%	Yes	13.16%
			No	47.00%	No	89.41%	No	86.84%
					Yes	4.84%		
					No	95.16%		
	No	79.84%	Yes	54.64%	Yes	6.94%	Yes	4.26%
			No	45.36%	No	93.06%	No	95.74%
					Yes	4.00%		
					No	96.00%		
	Fraction of Interacting Partners Retained After 3R		Fraction of Interatcing Partners Retained After 4R		Fraction of Interacting Partners Not Retained Ater 3R		Fraction of Interacting Partners not retained at 4R	
	38.33%		75.00%		35.76%		73.92%	
	Fraction of proteins with no interacting partners retained at 3R		Fraction of proteins with no interacting partners retained at 4R					
	35.62%		65.27%					

Salmon Duplication Analysis	is 3R Present?	Conditional Probabilities	is 4R Present?	Conditional Probabilities	is SSR Present?	Conditional Probabilities	is Pre4R Present?	Conditional Probabilities
Phylogeny relaxation with high thresholds of percent identy 50% and fraction of gaps 50%	Yes	18.81%	Yes	54.31%	Yes	7.50%	Yes	9.68%
			No	45.69%	No	92.50%	No	90.32%
					Yes	3.22%		
					No	96.78%		
	No	81.19%	Yes	51.80%	Yes	3.94%	Yes	2.78%
			No	48.20%	No	96.06%	No	97.22%
					Yes	2.33%		
					No	97.67%		
	Fraction of Interacting Partners Retained After 3R		Fraction of Interatcing Partners Retained After 4R		Fraction of Interacting Partners Not Retained Ater 3R		Fraction of Interacting Partners not retained at 4R	
	34.13%		68.42%		31.67%		68.33%	
	Fraction of proteins with no interacting partners retained at 3R		Fraction of proteins with no interacting partners retained at 4R					
	33.68%		64.17%					

Salmon Duplication Analysis	is 3R Present?	Conditional Probabilities	is 4R Present?	Conditional Probabilities	is SSR Present?	Conditional Probabilities	is Pre4R Present?	Conditional Probabilities
Strict phylogeny with no threshold	Yes	20.16%	Yes	45.99%	Yes	11.61%	Yes	18.66%
			No	54.01%	No	88.39%	No	81.34%
					Yes	4.02%		
					No	95.98%		
	No	79.84%	Yes	47.44%	Yes	7.61%	Yes	9.31%
			No	52.56%	No	92.39%	No	90.69%
					Yes	3.30%		
					No	96.70%		
	Fraction of Interacting Partners Retained After 3R		Fraction of Interatcing Partners Retained After 4R		Fraction of Interacting Partners Not Retained Ater 3R		Fraction of Interacting Partners not retained at 4R	
	38.33%		69.27%		35.76%		68.59%	
	Fraction of proteins with no interacting partners retained at 3R		Fraction of proteins with no interacting partners retained at 4R					
	35.62%		60.95%					

Salmon Duplication Analysis	is 3R Present?	Conditional Probabilities	is 4R Present?	Conditional Probabilities	is SSR Present?	Conditional Probabilities	is Pre4R Present?	Conditional Probabilities
Strict phylogeny with threshold percent identy 50% and fraction of gaps 50%	Yes	18.81%	Yes	46.81%	Yes	8.25%	Yes	15.40%
			No	53.19%	No	91.75%	No	84.60%
					Yes	2.63%		
					No	97.37%		
	No	81.19%	Yes	44.94%	Yes	4.34%	Yes	7.63%
			No	55.06%	No	95.66%	No	92.37%
					Yes	1.95%		
					No	98.05%		
	Fraction of Interacting Partners Retained After 3R		Fraction of Interatcing Partners Retained After 4R		Fraction of Interacting Partners Not Retained Ater 3R		Fraction of Interacting Partners not retained at 4R	
	34.13%		62.88%		31.67%		61.91%	
	Fraction of proteins with no interacting partners retained at 3R		Fraction of proteins with no interacting partners retained at 4R					
	33.68%		58.78%					

doi:10.1038/nature17164

All scripts used in this analysis will be made freely available on the Liberles Group website at Temple University (USA) at https://liberles.cst.temple.edu/public/Salmon_Genome_Project/.

9. Reference genome for salmonids

Construction of rainbow trout chromosome sequences. We used the Atlantic salmon assembly to construct chromosome sequences for the fragmented and non-chromosome anchored rainbow trout genome sequence[21]. Salmon chromosome sequences were repeat masked using a salmon repeat database (ssal_repeats_v2.0) and RepeatMasker v4.0.3[23] and aligned against rainbow trout scaffolds[21] using megablast[22]. Initially rainbow trout scaffolds mapping to multiple salmon chromosomes were broken when supported by information from a rainbow trout linkage map containing 31,390 SNPs (unpublished) constructed in a family material of 2,464 individuals using Lep-MAP[64]. Following breakage, the relative alignment of the rainbow trout scaffolds with the salmon genome and the rainbow trout linkage map were used to position, orientate and concatenate 11,335 rainbow trout scaffolds into 29 single chromosome sequences. In cases of discrepancy between the rainbow trout linkage map and the salmon genome assembly, the rainbow trout genome sequence was changed to match up with the linkage map. Total length of chromosome sequences was 1.37 Gb. Nomenclature for rainbow trout chromosomes followed[17].

Comparative mapping. Conserved syntenic blocks between rainbow trout and salmon were determined by aligning chromosome sequences for the two species against each other using LASTZ[25]. LASTZ command line script; --targetcapsule=LZ_target_capsule query.fa —nochain --gfextend --nogapped —identity=90.0..100.0 —matchcount=100 —format=general —rdotplot=plotoutput.txt. Alignment of these rainbow trout chromosomes with the Atlantic salmon genome revealed conservation of very large syntenic blocks in many cases corresponding to whole chromosome arms in rainbow trout (Extended Data Figure 1 and 10). Our analysis documents that the syntenic regions typically represent blocks with no rearrangements for 38 regions and with only one or two inversions/translocations among the remaining parts. We also identified two smaller syntenic blocks on ssa15qa-omy11q and ssa19qa-omy20p previously not reported, but they represent only a minor fraction of the genome.

The salmon - rainbow trout comparative map is presented in Extended Data Figure 1.

doi:10.1038/nature17164

References

1. Komen, H. & Thorgaard, G. H. Androgenesis, gynogenesis and the production of clones in fishes: a review. *Aquaculture* (2007). doi:10.1016/j.aquaculture.2007.05.009
2. Kodama, Y., Shumway, M., Leinonen, R.International Nucleotide Sequence Database Collaboration. The Sequence Read Archive: explosive growth of sequencing data. *Nucleic acids research* **40,** D54–6 (2012).
3. Osoegawa, K., de Jong, P. J., Frengen, E. & Ioannou, P. A. Construction of bacterial artificial chromosome (BAC/PAC) libraries. *Curr Protoc Hum Genet* **Chapter 5,** Unit 5.15 (2001).
4. Jaffe, D. B. *et al.* Whole-genome sequence assembly for mammalian genomes: Arachne 2. *Genome Res* **13,** 91–96 (2003).
5. Simpson, J. T. *et al.* ABySS: a parallel assembler for short read sequence data. *Genome Res* **19,** 1117–1123 (2009).
6. Huang, X., Wang, J., Aluru, S., Yang, S.-P. & Hillier, L. PCAP: a whole-genome assembly program. *Genome Res* **13,** 2164–2170 (2003).
7. Gnerre, S. *et al.* High-quality draft assemblies of mammalian genomes from massively parallel sequence data. *Proceedings of the National Academy of Sciences* **108,** 1513–1518 (2011).
8. Myers, E. W., Sutton, G. G., Delcher, A. L., Dew, I. M. & Fasulo, D. P. A whole-genome assembly of Drosophila. *Science (New York, NY)* (2000). doi:10.1126/science.287.5461.2196
9. Miller, J. R. *et al.* Aggressive assembly of pyrosequencing reads with mates. *Bioinformatics* **24,** 2818–2824 (2008).
10. Koren, S. *et al.* Hybrid error correction and de novo assembly of single-molecule sequencing reads. *Nature biotechnology* **30,** 693–700 (2012).
11. Zimin, A. V. *et al.* The MaSuRCA genome assembler. *Bioinformatics* **29,** 2669–2677 (2013).
12. Marcais, G. & Kingsford, C. A fast, lock-free approach for efficient parallel counting of occurrences of k-mers. *Bioinformatics* **27,** 764–770 (2011).
13. Zimin, A. V., Smith, D. R., Sutton, G. & Yorke, J. A. Assembly reconciliation. *Bioinformatics* **24,** 42–45 (2008).
14. Lien, S. *et al.* A dense SNP-based linkage map for Atlantic salmon (Salmo salar) reveals extended chromosome homeologies and striking differences in sex-specific recombination patterns. *BMC Genomics* **12,** 615 (2011).
15. Boetzer, M., Henkel, C. V., Jansen, H. J., Butler, D. & Pirovano, W. Scaffolding pre-assembled contigs using SSPACE. *Bioinformatics* **27,** 578–579 (2011).
16. Chaisson, M. J. & Tesler, G. Mapping single molecule sequencing reads using basic local alignment with successive refinement (BLASR): application and theory. *BMC Bioinformatics* **13,** 238 (2012).
17. Phillips, R. B. *et al.* Assignment of Atlantic salmon (Salmo salar) linkage groups to specific chromosomes: conservation of large syntenic blocks corresponding to whole chromosome arms in rainbow trout (Oncorhynchus mykiss). *BMC Genetics* **10,** 46 (2009).
18. Adzhubei, A. A. *et al.* Annotated expressed sequence tags (ESTs) from pre-smolt Atlantic salmon (Salmo salar) in a searchable data resource. *BMC Genomics* **8,** 209 (2007).
19. Koop, B. F. *et al.* A salmonid EST genomic study: genes, duplications, phylogeny and microarrays. *BMC Genomics* **9,** 545 (2008).
20. Leong, J. S. *et al.* Salmo salar and Esox lucius full-length cDNA sequences reveal changes in evolutionary pressures on a post-tetraploidization genome. *BMC Genomics* **11,** 279 (2010).
21. Berthelot, C. *et al.* The rainbow trout genome provides novel insights into evolution after whole-genome duplication in vertebrates. *Nat Comms* **5,** 3657 (2014).
22. Altschul, S. F., Gish, W., Miller, W., Myers, E. W. & Lipman, D. J. Basic local alignment search tool. *J. Mol. Biol.* **215,** 403–410 (1990).
23. Smit, A. F. A., Hubley, R. & Green, P. RepeatMasker Open-4.0. *www.repeatmasker.org* (2013). at <http://www.repeatmasker.org>
24. Parra, G., Bradnam, K., Ning, Z., Keane, T. & Korf, I. Assessing the gene space in draft genomes. *Nucleic acids research* **37,** 289–297 (2009).

25. Harris, R. S. *Improved Pairwise Alignment of Genomic DNA.* (ProQuest, 2007).
26. Matveev, V. & Okada, N. Retroposons of salmonoid fishes (Actinopterygii: Salmonoidei) and their evolution. *Gene* **434,** 16–28 (2009).
27. Jurka, J. *et al.* Repbase Update, a database of eukaryotic repetitive elements. *Cytogenet. Genome Res.* **110,** 462–467 (2005).
28. Flutre, T., Duprat, E., Feuillet, C. & Quesneville, H. Considering transposable element diversification in de novo annotation approaches. *PLoS ONE* **6,** e16526 (2011).
29. Smit, A. F. A. & Hubley, R. *RepeatModeler Open-1.0. www.repeatmasker.org* (2008). at <http://www.repeatmasker.org>
30. Ellinghaus, D., Kurtz, S. & Willhoeft, U. LTRharvest, an efficient and flexible software for de novo detection of LTR retrotransposons. *BMC Bioinformatics* **9,** 18 (2008).
31. Gremme, G., Brendel, V., Sparks, M. E. & Kurtz, S. Engineering a software tool for gene structure prediction in higher organisms. *Information and Software Technology* **47,** 965–978 (2005).
32. Jiang, N. Repeat Library Construction--Advanced. (2013). at <http://www.webcitation.org/6YWzgLCzw>
33. Steinbiss, S., Willhoeft, U., Gremme, G. & Kurtz, S. Fine-grained annotation and classification of de novo predicted LTR retrotransposons. *Nucleic acids research* **37,** 7002–7013 (2009).
34. Chan, P. P. & Lowe, T. M. GtRNAdb: a database of transfer RNA genes detected in genomic sequence. *Nucleic acids research* **37,** D93–7 (2009).
35. Camacho, C. *et al.* BLAST+: architecture and applications. *BMC Bioinformatics* **10,** 421 (2009).
36. Wicker, T. *et al.* A unified classification system for eukaryotic transposable elements. *Nat. Rev. Genet.* **8,** 973–982 (2007).
37. UniProt Consortium. UniProt: a hub for protein information. *Nucleic acids research* **43,** D204–12 (2015).
38. Quast, C. *et al.* The SILVA ribosomal RNA gene database project: improved data processing and web-based tools. *Nucleic acids research* **41,** D590–6 (2013).
39. Edgar, R. C. MUSCLE: multiple sequence alignment with high accuracy and high throughput. *Nucleic acids research* **32,** 1792–1797 (2004).
40. Tamura, K. *et al.* MEGA5: molecular evolutionary genetics analysis using maximum likelihood, evolutionary distance, and maximum parsimony methods. *Mol. Biol. Evol.* **28,** 2731–2739 (2011).
41. Pace, J. K., Gilbert, C., Clark, M. S. & Feschotte, C. Repeated horizontal transfer of a DNA transposon in mammals and other tetrapods. *Proceedings of the National Academy of Sciences* **105,** 17023–17028 (2008).
42. Bolger, A. M., Lohse, M. & Usadel, B. Trimmomatic: a flexible trimmer for Illumina sequence data. *Bioinformatics* **30,** 2114–2120 (2014).
43. Dobin, A. *et al.* STAR: ultrafast universal RNA-seq aligner. *Bioinformatics* **29,** 15–21 (2013).
44. Wu, T. D. & Nacu, S. Fast and SNP-tolerant detection of complex variants and splicing in short reads. *Bioinformatics* **26,** 873–881 (2010).
45. Haas, B. J. *et al.* De novo transcript sequence reconstruction from RNA-Seq: reference generation and analysis with Trinity. *Nat Protoc* **8,** 1494–1512 (2013).
46. Conesa, A. *et al.* Blast2GO: a universal tool for annotation, visualization and analysis in functional genomics research. *Bioinformatics* **21,** 3674–3676 (2005).
47. Katoh, K., Kuma, K.-I., Toh, H. & Miyata, T. MAFFT version 5: improvement in accuracy of multiple sequence alignment. *Nucleic acids research* **33,** 511–518 (2005).
48. Vigeland, M. D. *et al.* Evidence for adaptive evolution of low-temperature stress response genes in a Pooideae grass ancestor. *New Phytol.* **199,** 1060–1068 (2013).
49. Schliep, K. P. phangorn: phylogenetic analysis in R. *Bioinformatics* **27,** 592–593 (2011).
50. Yang, Z. PAML 4: phylogenetic analysis by maximum likelihood. *Mol. Biol. Evol.* **24,** 1586–1591 (2007).

51. Conant, G. C. & Wolfe, K. H. Turning a hobby into a job: how duplicated genes find new functions. **9,** 938–950 (2008).
52. Drummond, A. J., Suchard, M. A., Xie, D. & Rambaut, A. Bayesian phylogenetics with BEAUti and the BEAST 1.7. *Mol. Biol. Evol.* **29,** 1969–1973 (2012).
53. Macqueen, D. J., Garcia de la Serrana, D. & Johnston, I. A. Evolution of ancient functions in the vertebrate insulin-like growth factor system uncovered by study of duplicated salmonid fish genomes. *Mol. Biol. Evol.* **30,** 1060–1076 (2013).
54. Near, T. J. *et al.* Resolution of ray-finned fish phylogeny and timing of diversification. *Proceedings of the National Academy of Sciences* **109,** 13698–13703 (2012).
55. Rambaut, A., Suchard, M. A., Xie, D. & Drummond, A. J. Tracer v1.6. (2014). at <http://beast.bio.ed.ac.uk/Tracer>
56. R Development Core Team. *R: A language and environment for statistical computing*. (R Foundation for Statistical Computing, 2009). at <http://www.R-project.org/>
57. Falcon, S. & Gentleman, R. Using GOstats to test gene lists for GO term association. *Bioinformatics* **23,** 257–258 (2007).
58. Cunningham, F. *et al.* Ensembl 2015. *Nucleic acids research* **43,** D662–9 (2015).
59. Katoh, K. & Standley, D. M. MAFFT multiple sequence alignment software version 7: improvements in performance and usability. *Mol. Biol. Evol.* **30,** 772–780 (2013).
60. Guindon, S. *et al.* New algorithms and methods to estimate maximum-likelihood phylogenies: assessing the performance of PhyML 3.0. *Systematic Biology* **59,** 307–321 (2010).
61. Berglund-Sonnhammer, A.-C., Steffansson, P., Betts, M. J. & Liberles, D. A. Optimal gene trees from sequences and species trees using a soft interpretation of parsimony. *J. Mol. Evol.* **63,** 240–250 (2006).
62. Jensen, L. J. *et al.* STRING 8--a global view on proteins and their functional interactions in 630 organisms. *Nucleic acids research* **37,** D412–6 (2009).
63. Agresti, A. *Categorical Data Analysis*. *Categorical Data Analysis* (John Wiley & Sons, Inc., 2002). doi:10.1002/0471249688
64. Rastas, P., Paulin, L., Hanski, I., Lehtonen, R. & Auvinen, P. Lep-MAP: fast and accurate linkage map construction for large SNP datasets. *Bioinformatics* **29,** 3128–3134 (2013).
