# Supplementary material for: The Atlantic salmon genome provides insights into rediploidization
Source: Nature. 2016 Apr 18;533(7602):200–5. doi: 10.1038/nature17164 (PMC8127823; doi:10.1038/nature17164)
Supplement: Supplementary file 5 — PowerPoint slide for Fig. 1 [file 41586_2016_BFnature17164_MOESM5_ESM.ppt]

## Slide 1
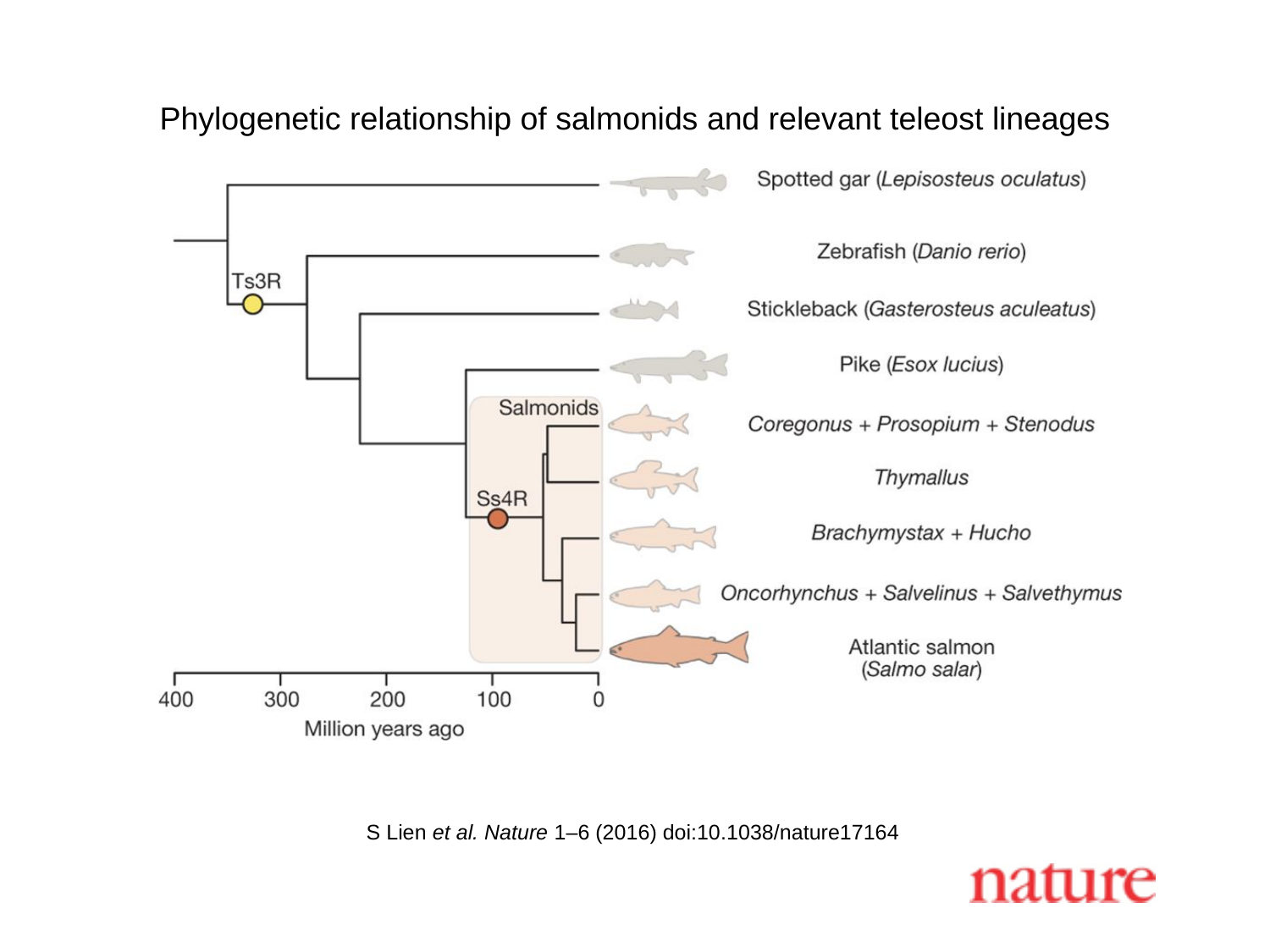

# Phylogenetic relationship of salmonids and relevant teleost lineages
S Lien et al. Nature 1–6 (2016) doi:10.1038/nature17164
