# Supplementary figures and images for: The Atlantic salmon genome provides insights into rediploidization
Source: Nature. 2016 Apr 18;533(7602):200–5. doi: 10.1038/nature17164 (PMC8127823; doi:10.1038/nature17164)

## Slide 1
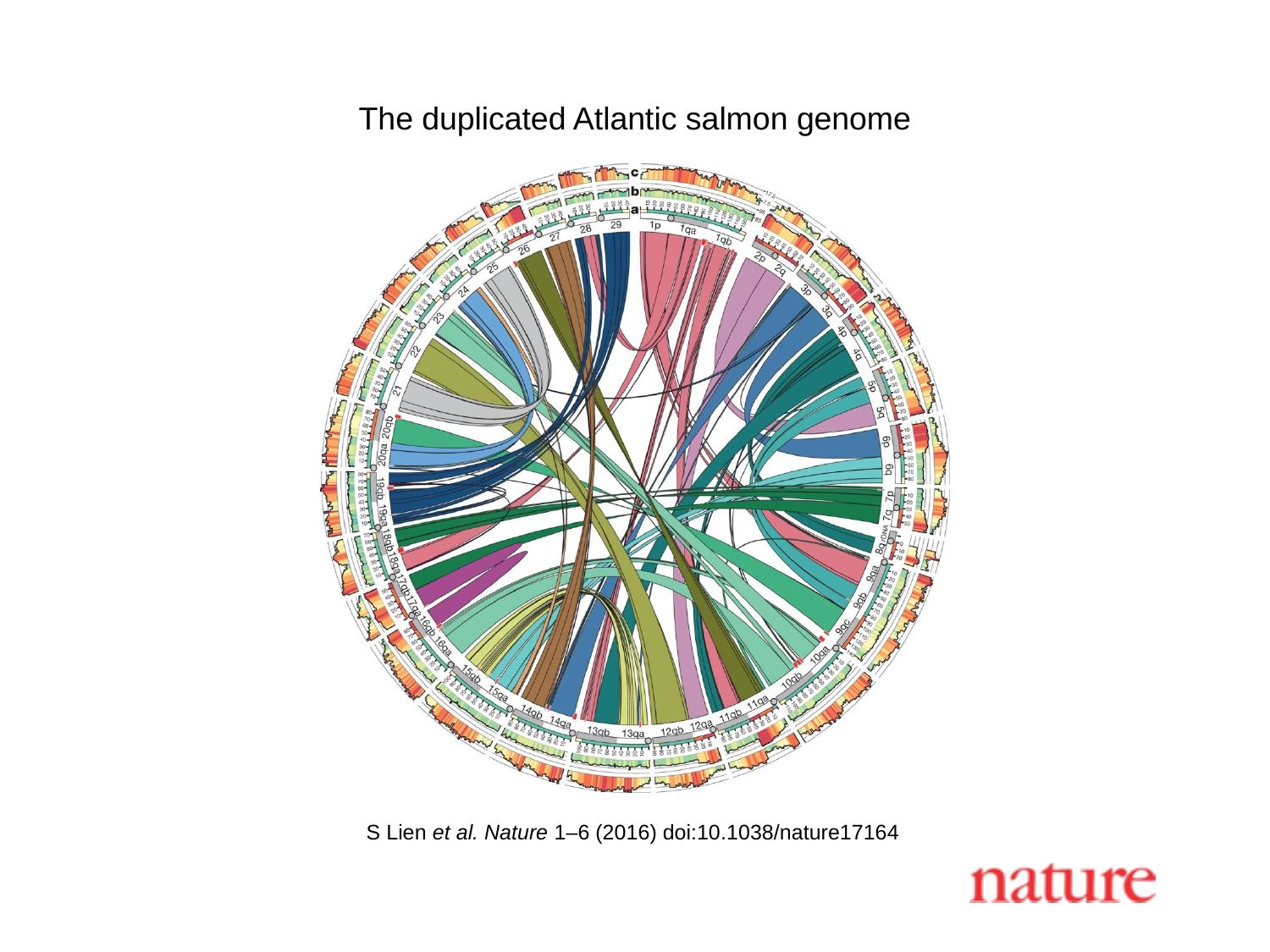

# The duplicated Atlantic salmon genome
S Lien et al. Nature 1–6 (2016) doi:10.1038/nature17164

Supplement: Supplementary file 6 — PowerPoint slide for Fig. 2 [file 41586_2016_BFnature17164_MOESM6_ESM.ppt]

## Slide 1
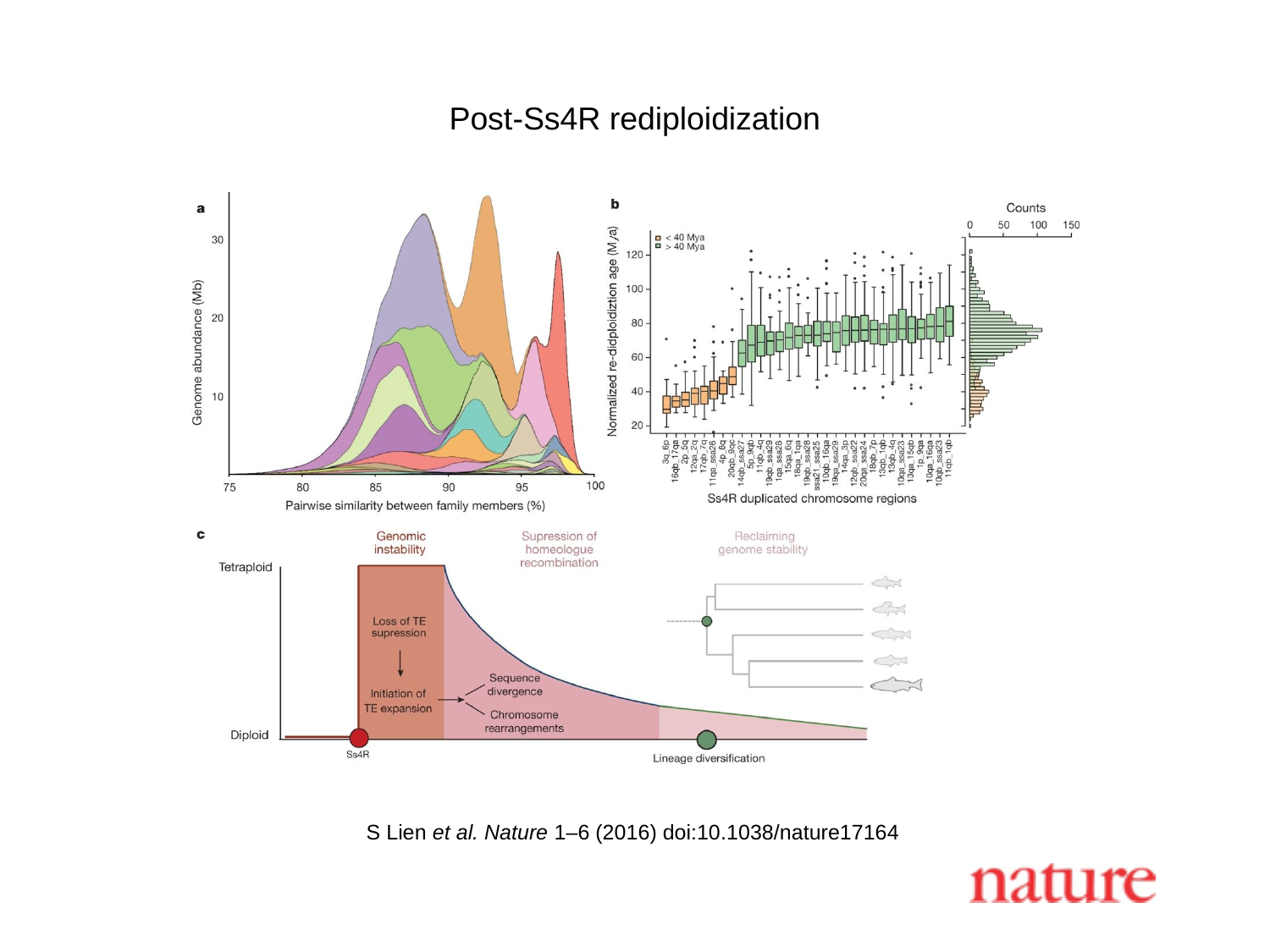

# Post-Ss4R rediploidization
S Lien et al. Nature 1–6 (2016) doi:10.1038/nature17164

Supplement: Supplementary file 7 — PowerPoint slide for Fig. 3 [file 41586_2016_BFnature17164_MOESM7_ESM.ppt]

## Slide 1
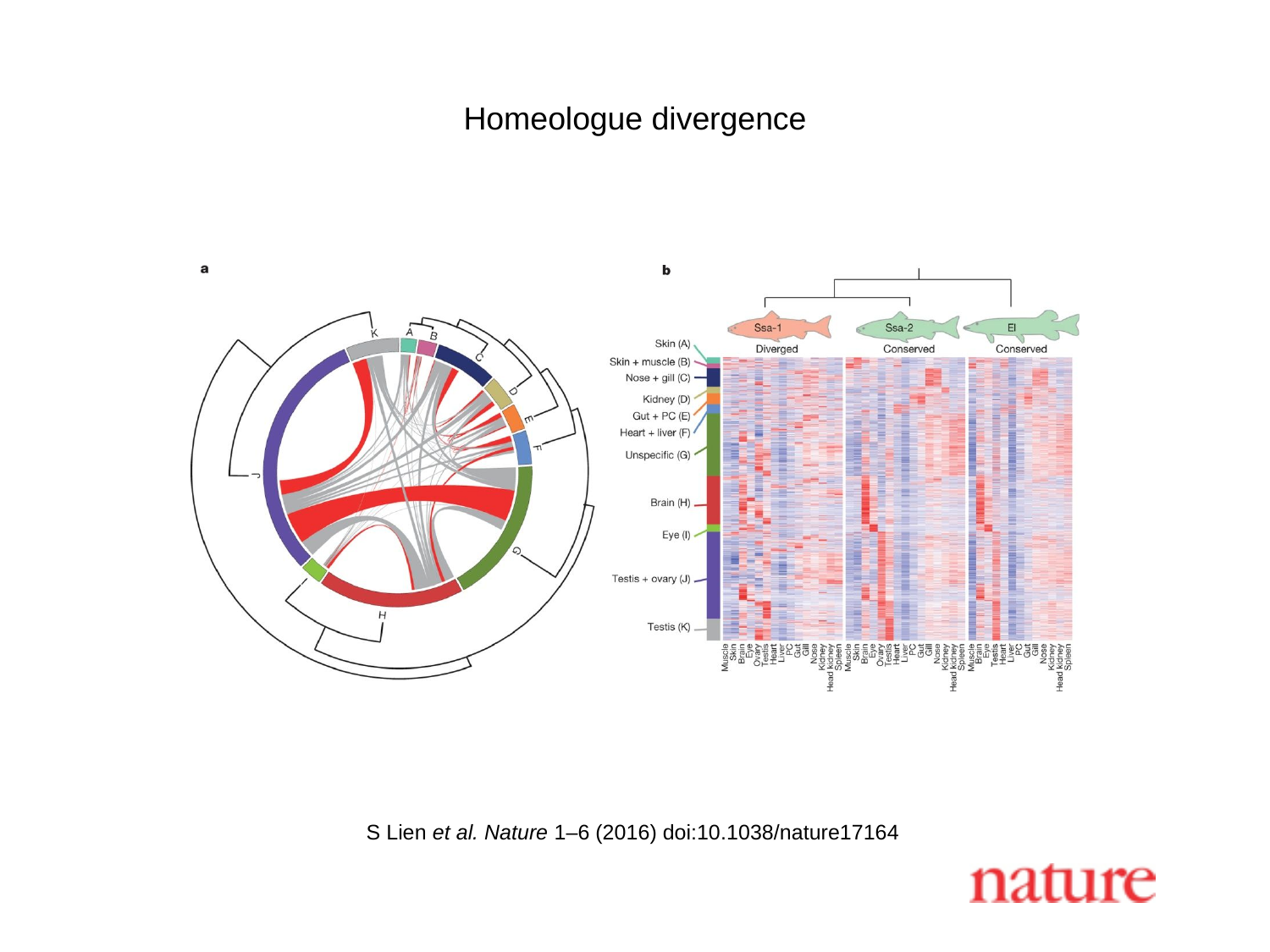

# Homeologue divergence
S Lien et al. Nature 1–6 (2016) doi:10.1038/nature17164

Supplement: Supplementary file 8 — PowerPoint slide for Fig. 4 [file 41586_2016_BFnature17164_MOESM8_ESM.ppt]
